# Contrasting Metallic (Rh^0^) and Carbidic (2D-Mo_2_C MXene) Surfaces in Olefin Hydrogenation Provides Insights on the Origin of the Pairwise Hydrogen Addition

**DOI:** 10.1021/acscatal.4c02534

**Published:** 2024-08-06

**Authors:** Ling Meng, Ekaterina V. Pokochueva, Zixuan Chen, Alexey Fedorov, Francesc Viñes, Francesc Illas, Igor V. Koptyug

**Affiliations:** † Departament de Ciència de Materials i Química Física & Institut de Química Teòrica i Computacional (IQTCUB), Universitat de Barcelona, c/Martí i Franquès 1-11, 08028 Barcelona, Spain; ‡ International Tomography Center SB RAS, 3A Institutskaya St., Novosibirsk 630090, Russian Federation; § Department of Mechanical and Process Engineering, ETH Zürich, Leonhardstrasse 21, Zürich 8092, Switzerland

**Keywords:** 2D-Mo_2_C, Rh^0^, MXenes, ethene, parahydrogen, pairwise hydrogenation

## Abstract

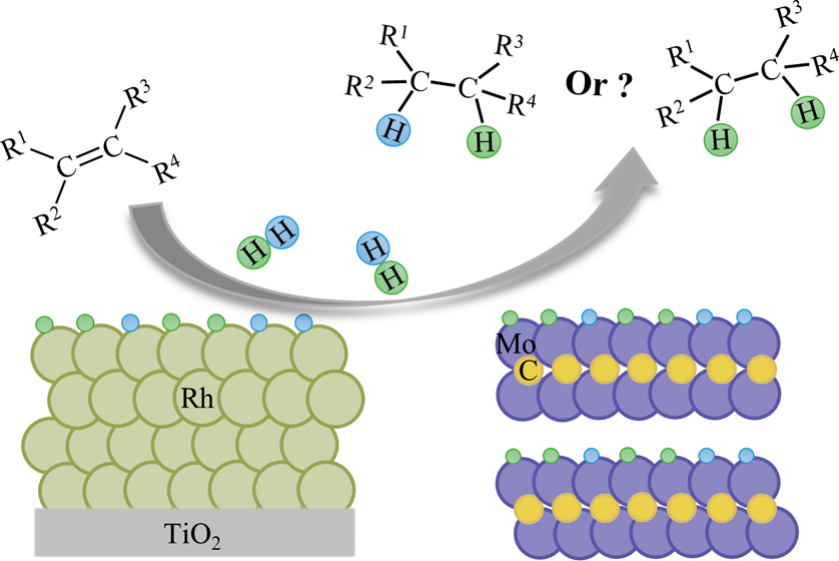

Kinetic studies are vital for gathering mechanistic insights into heterogeneously catalyzed hydrogenation of unsaturated organic compounds (olefins), where the Horiuti–Polanyi mechanism is ubiquitous on metal catalysts. While this mechanism envisions nonpairwise H_2_ addition due to the rapid scrambling of surface hydride (H*) species, a pairwise H_2_ addition is experimentally encountered, rationalized here based on density functional theory (DFT) simulations for the ethene (C_2_H_4_) hydrogenation catalyzed by two-dimensional (2D) MXene Mo_2_C(0001) surface and compared to Rh(111) surface. Results show that ethyl (C_2_H_5_*) hydrogenation is the rate-determining step (RDS) on Mo_2_C(0001), yet C_2_H_5_* formation is the RDS on Rh(111), which features a higher reaction rate and contribution from pairwise H_2_ addition compared to 2D-Mo_2_C(0001). This qualitatively agrees with the experimental results for propene hydrogenation with parahydrogen over 2D-Mo_2_C_1–*x*_ MXene and Rh/TiO_2_. However, DFT results imply that pairwise selectivity should be negligible owing to the facile H* diffusion on both surfaces, not affected by H* nor C_2_H_4_* coverages. DFT results also rule out the Eley–Rideal mechanism appreciably contributing to pairwise addition. The measurable contribution of the pairwise hydrogenation pathway operating concurrently with the dominant nonpairwise one is proposed to be due to the dynamic site blocking at higher adsorbate coverages or another mechanism that would drastically limit the diffusion of H* adatoms.

## 1 Introduction

**Scheme 1:**
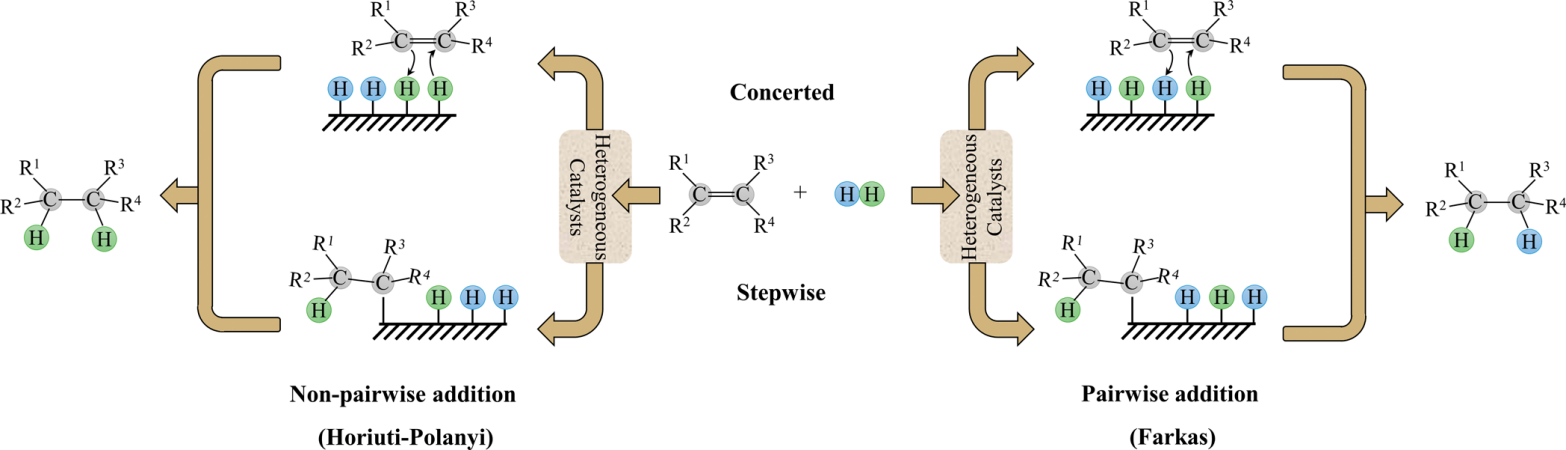
Horiuti–Polanyi (Nonpairwise) and Farkas (Pairwise) Hydrogen Addition Mechanisms

Hydrogenation reactions are at the core of heterogeneous catalysis, spanning from environmental treatments and the petrochemical industry to the synthesis of fine chemicals.^1^ Regardless of a particular application, the surface of a heterogeneous catalyst provides active sites for weakening the H_2_ bond, leading ultimately to the dissociative chemisorption that yields active surface H adatoms (H*).^2^ Subsequently, H* species are transferred to a substrate, *e.g.*, in the hydrogenation of unsaturated hydrocarbons such as alkynes and alkenes.^3^ The catalytic hydrogenation of alkenes has nearly a centenary-long research history, owing to a high practical relevance.^4^ A textbook example is the hydrogenation of ethene, *i.e.*, C_2_H_4_^(g)^ + H_2_^(g)^ → C_2_H_6_^(g)^, perhaps, the most extensively studied alkene hydrogenation reaction. From the mechanism proposed by Horiuti and Polanyi (*cf.*Scheme 1), which is widely recognized as the prevalent route for alkene hydrogenation,^5,6^ it follows that the addition of H_2_ to ethene is nonpairwise, *i.e.*, the added H atoms generally come from different H_2_ molecules. In the Horiuti–Polanyi mechanism, the rapid surface diffusion of the H* species plays a crucial role in randomly adding hydrogen atoms to a substrate (ethene).^7^

That being said, it is now conclusively established that various types of heterogeneous catalysts can achieve pairwise hydrogen addition, whereby the two H atoms that end up in the hydrogenation product molecule originate from the same H_2_ molecule.^8^ The unambiguous evidence for this is provided by experiments that use parahydrogen (*p*-H_2_), the nuclear spin isomer of H_2_ with the opposite spin orientation of its two H atoms (more rigorously, it is a state with zero total nuclear spin). The addition of *p*-H_2_ to various alkenes or alkynes leads, when the addition proceeds in a pairwise manner, to a nonequilibrium population of nuclear spin states in the product(s), resulting in a major (*i.e.*, orders of magnitude) signal enhancement in NMR spectra of the reaction products. Parahydrogen-induced polarization (PHIP) is a method that allows one to evaluate the selectivity of a catalyst to the pairwise addition of H_2_ as the detected NMR signal enhancement is directly proportional to the pairwise selectivity,^9,10^ aided by the high sensitivity of PHIP as a mechanistic tool that identifies reliably the contribution of the pairwise hydrogenation pathway of merely *ca.* 0.01%.^8^

Using the PHIP approach, various metal-based catalysts have been demonstrated to provide pairwise selectivity in an order of several percent. Pairwise addition of H_2_ has been observed for catalysts based on Rh,^11^ Pd,^12^ Pt,^13,14^ Ir,^15^ V,^16^ Cu,^10^ and Co,^17^ among others. Furthermore, the hexagonal close-packed (hcp) phase of Mo_2_C also exhibited a significant selectivity in the pairwise hydrogen addition, resulting in approximately a 150-fold increase in signal intensity, compared to the face-centered-cubic (fcc) MoC phase containing C-vacancies.^18^

These experiments involving p-H_2_ demonstrate unambiguously the feasibility of the pairwise addition on metal and metal carbide surfaces, which generally disagrees with the commonly accepted Horiuti–Polanyi mechanism that assumes a rapid diffusion of H* species.^4,19^ Yet, an alternative reaction route proposed by Farkas entails a slow diffusion of H* and allows for the pairwise addition pathway (*cf.*Scheme 1).^20^ Note that, in principle, both concerted and stepwise hydrogen additions can follow pairwise and nonpairwise routes, as presented in Scheme 1.^21^

The arguments above notwithstanding, it remains challenging to predict (and even rationalize) the selectivity preference to the pairwise hydrogen addition pathway for a given catalytic surface. To understand the interplay between the diffusion rate of H* species, the energy barriers on the hydrogenation pathway, and the contribution from the pairwise addition to the overall hydrogenation rate, we relied on the density functional theory (DFT) computations of a model ethene (C_2_H_4_) hydrogenation to ethane (C_2_H_6_). Rh was chosen for this study as it is one of the most active catalysts in the hydrogenation of unsaturated substrates, with Rh/TiO_2_ demonstrating pairwise selectivities of up to 8%.^11^ Metal carbides are also efficient hydrogenation catalysts and, as mentioned above, can produce pronounced PHIP effects. It was anticipated that the presence of carbide phases or carbidic species would affect the rate of hydrogenation and significantly modify the diffusive mobility of surface H* species. As the diffusive separation of H* species highly favors nonpairwise H_2_ addition, it was deemed instructive to consider such effects in this study. Given the known instability of Rh_2_C under hydrogenation conditions,^22^ 2D-Mo_2_C(0001) appears to be a more suitable carbide owing to its higher thermal stability (relative to noble metal carbides such as Rh_2_C or PdC_*x*_) and well-defined nature (the presence of single surface termination in MXenes). Consequently, we have explored a well-defined 2D-Mo_2_C MXene exhibiting predominantly the basal (0001) surface,^23^ and contrasted the results to those obtained for the Rh(111) surface. Both 2D-Mo_2_C and C-deficient 2D-Mo_2_C_1–*x*_ can be obtained experimentally by a reductive defunctionalization of Mo_2_C*T*_*x*_ (*T*_*x*_ are surface functional groups) of the MXene family.^24,25^ Worthy of note, hydrogenation reactions of unsaturated hydrocarbons on MXenes are largely understudied.^26^

To shed light on the origin of PHIP effects, we considered thermodynamic and kinetic aspects of H_2_ addition in both concerted and stepwise pathways when departing from H* as generated upon H_2_ adsorption and dissociation on the catalyst surface. Note that the energetics associated with the two mechanisms are almost identical. Thus, to ease the upcoming discussion, only the stepwise mechanism is presented in detail here, using a pool of diffusing H* adatoms on the 2D-Mo_2_C(0001) and the Rh(111) surfaces. The DFT predictions were then assessed experimentally using parahydrogen addition to propene on 2D-Mo_2_C_1–*x*_ and Rh/TiO_2_ catalysts.^27^ The use of a simpler ethene model instead of propene in the DFT calculations is supported by the observation that H_2_ activation mainly originates from the electrostatic potential and charge on metal sites on the catalyst surface, with only a limited influence from the substrate.^27^ Ethene and propene are homologous olefins with a single double bond and so a similar reactive site.^28^ The ethene hydrogenation rate was reported to be ca. an order of magnitude higher than that of propene,^29,30^ implying a difference of only 0.06–0.1 eV in the activation energy barrier according to the Arrhenius equation. Thus, using a simpler ethene structure reduces computational complexity and time without compromising the value of insights for propene hydrogenation. The study reveals that, upon H_2_ dissociation, H* species undergo diffusion before transferring to alkenes and emphasizes the effect of surface coverage (including hydrides and alkene adsorbates) on the rate of ethene hydrogenation (with only a minor effect found when considering 3/4 monolayer of H* or C_2_H_4_* species), demonstrating the vastly dominant nonpairwise mechanism for both Rh(111) and 2D-Mo_2_C(0001), regardless of a stacking motif (ABA or ABC). Overall, our results demonstrate that, within the range of surface coverages explored, the inherently nonpairwise nature of the Horiuti–Polanyi mechanism cannot be reconciled with the pronounced contribution of the pairwise hydrogenation pathway observed experimentally. Therefore, alternative possibilities for the pairwise H_2_ addition on the surface of heterogeneous catalysts need to be considered.

## 2 Experimental and Theoretical Aspects

### 2.1 Computational Details

Periodic DFT calculations were carried out using the Vienna *ab initio* simulation package (VASP).^31^ The exchange-correlation interaction was approximated within the generalized gradient approximation (GGA) using the formalism proposed by Perdew–Burke–Ernzerhof (PBE),^32^ including Grimme’s D3 approach to account for dispersive interactions.^33^ The projector-augmented wave (PAW) method,^34^ as implemented in VASP by Kresse and Joubert,^35^ was chosen to describe the density of core electrons and their effect on the valence electron density. The valence electron density was expanded on a plane wave basis set with a cutoff kinetic energy of 415 eV.

The Rh reference (111) surface, the most stable one of Rh,^36^ was modeled using a *p*(4 × 4) slab with four fully optimized layers constructed from the optimized geometry of Rh bulk, as described in ref (36). A vacuum region of at least 16 Å was used perpendicular to the surface direction to avert interactions between the periodically repeated models. Likewise, the 2D-Mo_2_C MXene(0001) basal surfaces, featuring either the regular ABC stacking, or the energetically more stable ABA stacking,^37^ were modeled using a *p*(4 × 4) supercell. For such models, an optimal 4 × 4 × 1***k***-point **Γ**-centered Monkhorst–Pack grid was used to sample the Brillouin zone for the necessary numerical integration in the reciprocal space.^38^ During the geometry optimization of the models, either pristine or with adsorbates, a convergence criterion of 10^–5^ eV was used for the electronic self-consistent field steps, while the relaxation of atomic positions finalized when forces acting on atoms were below 0.01 eV·Å^–1^. Based on previous calculations, differences in computational details, such as operation thresholds, ***k***-points densities, and basis set sizes, resulted in variations in total energy below the chemically meaningful precision threshold of *ca.* 0.04 eV.^39^

The obtained optimized structures of surface species on the reaction coordinate were characterized as minima via vibrational frequency analysis, gained Hessian matrix diagonalization involving adsorbate degrees of freedom only, with elements obtained from finite differences of analytical gradients with steps of 0.03 Å in length,^40^ thus assuming vibrational decoupling from surface phonons, following reported approaches.^41−43^ For each adsorbate, different high-symmetry adsorption sites were sampled, see Figure S1 of the Supporting Information (SI), and for each adsorbate, various orientations and connectivities were considered systematically. For each species, *i*, and for each found minimum, adsorption energies, Δ*E*_ads_^*i*^, were calculated as
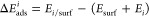
1where *E*_*i*/surf_ is the energy of the surface with the adsorbate, *E*_surf_ stands for the energy of the clean surface, *i.e.*, ABC- or ABA-stacked 2D-Mo_2_C MXene(0001) surface, or Rh(111) surface models, and *E*_*i*_ is the energy of the *i* species in the gas phase as optimized in vacuum considering the **Γ**-point only within a box of broken-symmetry dimensions of 9 × 10 × 11 Å^3^. Notice that, strictly speaking, such energies can be regarded as adsorption energies for the species existing in the gas phase, namely, H_2_, C_2_H_4_, and C_2_H_6_, while for H and C_2_H_5_, these would be interaction energies, but in what follows, they are discussed indistinctly as adsorption energies. Within this definition, the more negative the Δ*E*_ads_^*i*^, the stronger the interaction is. In addition, the adsorption Gibbs free energies at a given temperature, *T*, and gas partial pressure, *p*, were gained. The details are provided in Section S1 of the SI.

Once reactants are adsorbed, and as far as the stepwise ethene hydrogenation mechanism is concerned, we regarded the following reaction steps

(i) H_2_ dissociation, Δ*E*_diss_:
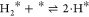
2

(ii) First hydrogen transfer, Δ*E*_H_^1st^:
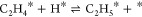
3

(iii) Second hydrogen transfer, Δ*E*_H_^2st^:
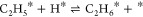
4including also adsorption and desorption steps of H_2_, C_2_H_4_, and C_2_H_6_. In addition, diffusion of surface species has been investigated. For the reactive and diffusive paths, transition states (TSs) have been located using the climbing-image nudged elastic band (CI-NEB) and the improved dimer approaches,^44,45^ applied on the most stable structures of initial states (ISs) and final states (FSs). As was described above for the identified minima, the TSs are characterized as well as saddle points, thus featuring zero gradients and a single imaginary frequency, consistent with the reaction path. Adsorption, desorption, diffusive, and reactive rates have been obtained as detailed in Section S2 of the SI. The span model used is described in Section S3 of the SI.

### 2.2 Experimental Details

Rh/TiO_2_ with 1 wt % of Rh (determined with an X-ray fluorescence method) was prepared by the incipient impregnation method. Before use, TiO_2_ (Hombifine N, phase-pure anatase, *S*_BET_ = 103 m^2^ g^–1^) was calcined at 550 °C for 2 h and dried at 120 °C for 6 h prior to impregnation. The support was impregnated with a solution of rhodium(III) nitrate (Sigma-Aldrich), thoroughly mixed, and left in air for 24 h, followed by drying in air for 3 h at 120 °C, calcination in air for 2 h at 600 °C and a treatment with H_2_ for 3 h at 330 °C. The size of Rh particles in the resulting Rh/TiO_2_ was assessed with transmission electron microscopy and was found to be *ca.* 1.4 nm. The particle dispersion, calculated from CO chemisorption measurements, was 77%, consistent with the previous report.^11^ Mo_2_C*T*_*x*_ was obtained by etching Mo_2_Ga_2_C with concentrated hydrofluoric acid (HF) as described previously.^24,46,47^ The same batch of Mo_2_C*T*_*x*_ was used for experiments in this work, as reported in ref (24). For the hydrogenation experiments, 100 mg of Mo_2_C*T*_*x*_ was loaded in a 1:4″ OD stainless steel reactor and held in place using two pieces of a fiberglass tissue. Hydrogen gas was enriched with parahydrogen up to *ca.* 95% using a *p*-H_2_ generator based on a closed-cycle helium cryostat (Cryotrade engineering CryoPribor, model CFA-200-H2cell) and a cryo-compressor (Vacree Technologies Co., Ltd., model C100A). Propene and *p*-H_2_ were supplied separately through Bronkhorst mass-flow controllers and were mixed directly in the gas lines with a volume ratio of 1:4. The resulting mixture was supplied through a 1:16″ OD PTFE capillary via a Y-connector from PEEK polymer to the reactor, and then to the 10 mm NMR tube placed inside the NMR spectrometer (300 MHz). A valve added between the line from the reactor and a bypass line allowed for a facile acquisition of NMR spectra with the complete nuclear spin relaxation without termination of the gas flow through the catalyst layer. The reactor was heated with a tubular furnace. The Mo_2_C*T*_*x*_ precursor was pretreated in an H_2_ flow of 60 mL min^–1^ at 500 °C for 2 h (heating ramp was 10 °C min^–1^), to give a material denoted as Mo_2_C*T*_*x*–500_, and then cooled down to 150 °C without termination of the gas flow. Subsequently, the catalyst was tested in the temperature range from 165 to 375 °C and at a gas flow rate of 26, 156, and 240 mL min^–1^. Alternatively, 5 mg of Rh/TiO_2_ was mixed with 20 mg of SiC (*S*_BET_ ca. 6 m^2^ g^–1^) and pretreated before the catalytic test in an H_2_ flow of 30 mL min^–1^ at 200 °C for 1 h, then cooled down to 43 °C without termination of the gas flow. In this case, the catalytic test was performed in the temperature range from 43 to 150 °C using the same gas flow rates of 26, 156, and 240 mL min^–1^.

Conversion of propene (X_C_3_H_6__) was calculated from the ratio of the integral of propane (*i.e.*, hydrogenation product) NMR signal (*S*_C_3_H_8__) to the sum of the integrals of propane and unreacted propene (*i.e.*, substrate) signals (*S*_C_3_H_6__), determined from the spectra acquired after relaxation of nuclear spins to the thermal equilibrium
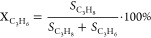
5

Signal enhancement (SE) for CH_3_-groups of propane was evaluated as
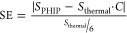
6where *S*_PHIP_ is the integral of the NMR signal of hyperpolarized propane (acquired during gas flow), *S*_thermal_ is the integral of the NMR signal of propane after relaxation to thermal equilibrium, and 6 is the number of protons in the two CH_3_ groups of propane. *C* is the coefficient of NMR signal suppression at high flow rates caused by a fast inflow of reagents from the Earth’s magnetic field to the NMR probe due to an insufficient time for the nuclear magnetization to achieve its high-field equilibrium value. *S*_thermal_ is evaluated after complete thermalization and is thus not affected by flow, whereas its contribution to the enhanced NMR signal is reduced by flow, *i.e.*, *C* < 1.

## 3 Results and Discussion

### 3.1 Computational Assessment

**Figure 1:**
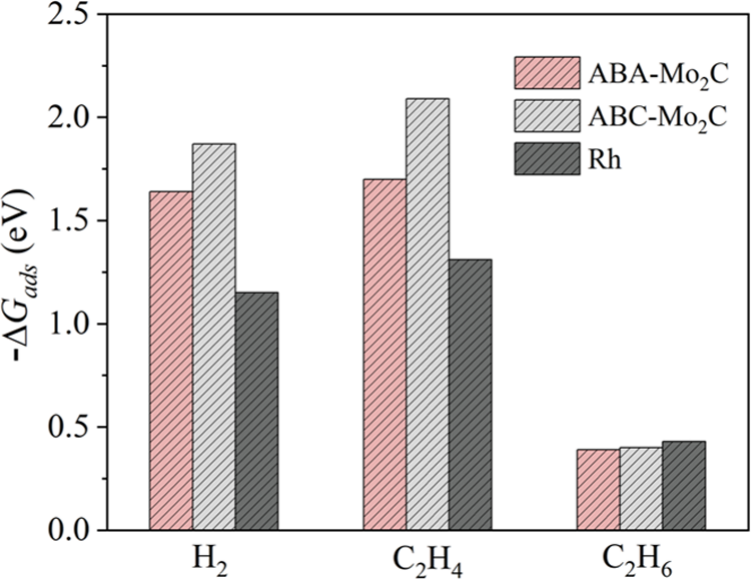
Adsorption free energies of H_2_ (assuming the spontaneous dissociation into 2H*), C_2_H_4_, and C_2_H_6_ on the ABA-Mo_2_C, ABC-Mo_2_C and Rh(111) surfaces under 1 bar of gas pressure and 250 or 60 °C for the 2D-Mo_2_C and Rh(111) models, respectively.

The reactants (C_2_H_4_* and H_2_*), the product (C_2_H_6_*), and the intermediates (H* and C_2_H_5_*) have been evaluated on high symmetry sites of the ABC- or ABA-stacked 2D-Mo_2_C(0001) surface, as well as on the Rh(111) surface, and the optimal adsorption sites are presented in Figure S2. The observed H_2_* minima on the 2D-Mo_2_C MXene model are in line with the previous report.^48^ The adsorption Gibbs free energies, Δ*G*_ads_, for reactants and product, plotted in Figure 1—values are provided in Table S1—reveal that, regardless of the stacking, the Δ*G*_ads_ energies of MXene-derived 2D-Mo_2_C are comparable to that of Rh, even though there are certain differences, *i.e.*, with the exception of C_2_H_6_, the Δ*G*_ads_ energies are larger on the 2D-Mo_2_C models relative to Rh(111). Among the 2D-Mo_2_C models, the Gibbs free energies of adsorption are larger for ABC-Mo_2_C than for ABA-Mo_2_C, consistent with a lower stability of the ABC-stacking relative to the ABA-stacking.^37^

**Figure 2:**
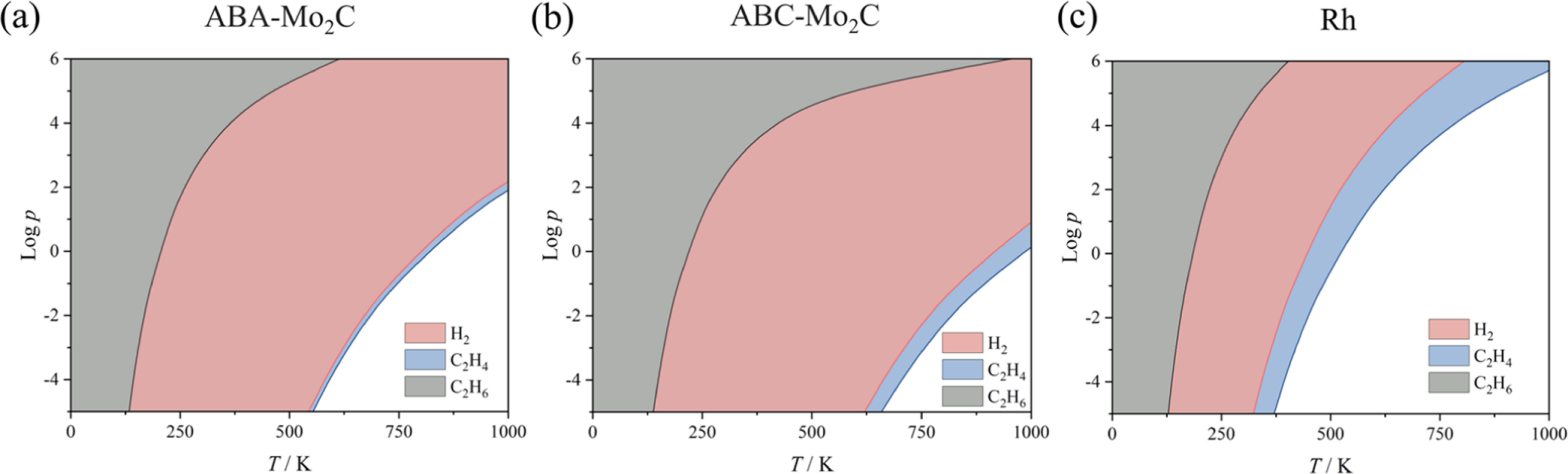
Calculated kinetic phase diagrams for H_2_, C_2_H_4_, and C_2_H_6_ on the (a) ABA-Mo_2_C, (b) ABC-Mo_2_C, and (c) Rh(111) models as a function of temperature *T*, in K, and standard logarithmic function of the gas pressures, *p*, in Pa. Colored regions imply a preference toward adsorption, while white areas represent regions where pristine surfaces are preferred.

The computed adsorption energies were used to estimate adsorption and desorption rates as a function of the gas pressure, *p*, and temperature, *T* (*cf.*Figure S3). In turn, the rates can be used to derive the so-called kinetic phase diagrams (KPD),^43^ presented in Figure 2. Given the easiness of H_2_* dissociation, see below, the formation of H_2_* from 2 H* and its subsequent desorption have been used for the KPD. With this in mind, a slightly higher affinity of the surfaces for C_2_H_4_ is found with respect to H_2_. It is clear that reactants, C_2_H_4_ and H_2_ (undergoing the dissociation to 2 H*), can be adsorbed on all three surfaces at the working conditions, while C_2_H_6_ would be prone to facile desorption; a favorable feature for the catalyst performance.

As briefly introduced, the dissociation of one H_2_* into 2 H* on the studied surfaces was assessed also in the vicinity of a single C_2_H_4_* species. H* adsorbs preferentially on the H_B_ and H_M_ sites for the ABA-Mo_2_C and ABC-Mo_2_C models, respectively (*cf.*Figure S1). However, on Rh(111), despite H_fcc_ being the most stable site for the H* adsorption, the adsorption of H* on H_hcp_ is energetically less exothermic by merely 0.03 eV (*cf.*Figure S1), implying both sites compete for the H* species. The number of conceivable intermediates for two vicinal H* is larger for the Rh(111) surface; see Figure S4 and Table S2 for the coadsorption energies. Competitive minima are used as final states for the H_2_* dissociation (*cf.*Figure S5), with the estimated dissociation energy barriers, *E*_b_, of only 0.28 and 0.11 eV for the ABA- and ABC-Mo_2_C models, respectively, and only 0.06 eV for Rh(111)—toward vicinal H_fcc_ and H_hcp_ sites—, and 0.08 eV toward two vicinal H_fcc_ sites. Thus, H_2_ dissociation is a low-energy barrier elementary step on all three pristine surfaces and slightly more difficult on 2D-Mo_2_C(0001) than on Rh(111).

Considering the notably stronger interaction of C_2_H_4_ compared to H_2_ molecule on all three surfaces, one can anticipate the dissociation of H_2_ to proceed also in the presence of C_2_H_4_*. To this end, the H_2_* adsorption sites and 2 H* coadsorption sites were probed, see Figures S6 and S7 for the respective structures, and Tables S3–S6 for the adsorption energies. The presence of C_2_H_4_* has only a moderate impact on the H_2_ dissociation energy barriers, *E*_b_ (cf. Table S7). In the presence of C_2_H_4_*, the *E*_b_ values decrease to 0.19 and 0.09 eV for ABA- and ABC-Mo_2_C, respectively, and to only 0.01 eV for the Rh(111) surface—ISs, TSs, and FSs corresponding to the paths with the lowest *E*_b_ are presented in Figure S8. To summarize, 2D-Mo_2_C and Rh(111) dissociate H_2_ easily, regardless of the presence or absence of the C_2_H_4_* species.

As aforementioned, the accepted mechanisms for the hydrogenation of alkenes over heterogeneous transition metal catalysts involve the dissociative chemisorption of H_2_.^49^ Upon H_2_ dissociation and prior to the H* transfer to an unsaturated hydrocarbon such as an alkene, the diffusion of H* adatoms may take place.^50^ The diffusion of H* adatoms was computationally explored considering the presence or absence of C_2_H_4_*, following the paths depicted in Figure S1, and, in the Rh(111) case, involving both H_fcc_ and H_hcp_ competitive sites. The preferred paths on the pristine surfaces, shown in Figure S9, reveal diffusion energy barriers *E*_b_ of 0.35 and 0.27 eV for the ABA- and ABC-Mo_2_C models and of 0.16 eV for the Rh(111) surface; these values vary slightly in the presence of C_2_H_4_* (cf. Figure S10), with the *E*_b_ values of 0.37 and 0.28 eV for the ABA- and ABC-Mo_2_C models, and 0.10 eV for Rh(111). Altogether, the results show that regardless of the absence or presence of C_2_H_4_*, the *E*_b_ of H_2_ dissociation is lower than that of H* diffusion (cf. Table S8).

**Figure 3:**
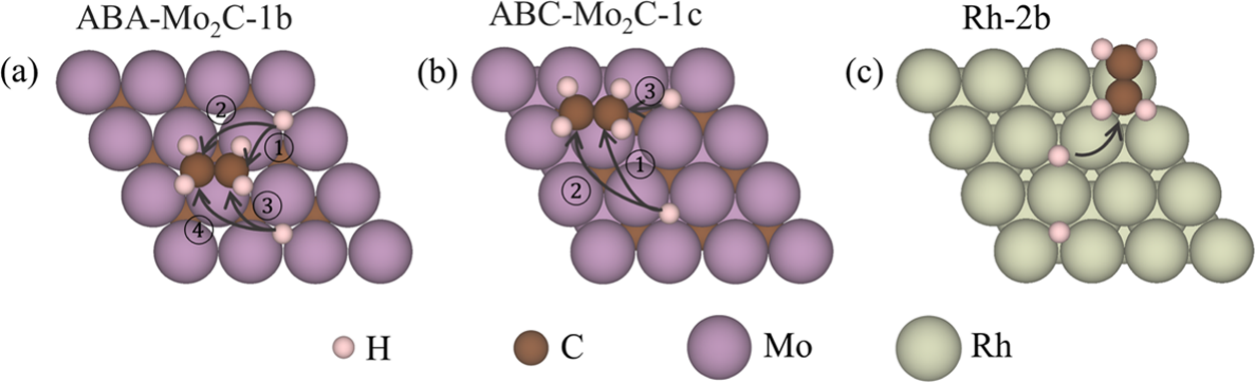
Potential routes of the first step of the C_2_H_4_ hydrogenation reaction on (a) ABA-Mo_2_C-1b, (b) ABC-Mo_2_C-1c, and (c) Rh-2b. See Figure S7 for the definition of notations.

Next, the energetics of the ethene hydrogenation steps were evaluated. The reactive pathways for the first H* transfer are shown in Figure 3, while reaction energy changes, Δ*E*, and energy barriers, *E*_b_, are presented in Table S9. The Δ*E* to form C_2_H_5_* from C_2_H_4_* and H* on the 2D-Mo_2_C models range from 0.28 to 0.57 eV, which is generally comparable to the Δ*E* of 0.39 eV on Rh(111). Similarly, the *E*_b_ range from 0.64 to 0.84 eV on the 2D-Mo_2_C models, which are slightly lower than 0.91 eV found for Rh(111). Thus, the first H* transfer step to C_2_H_4_* is more facile on 2D-Mo_2_C regardless of the stacking when compared to Rh(111).

**Figure 4:**
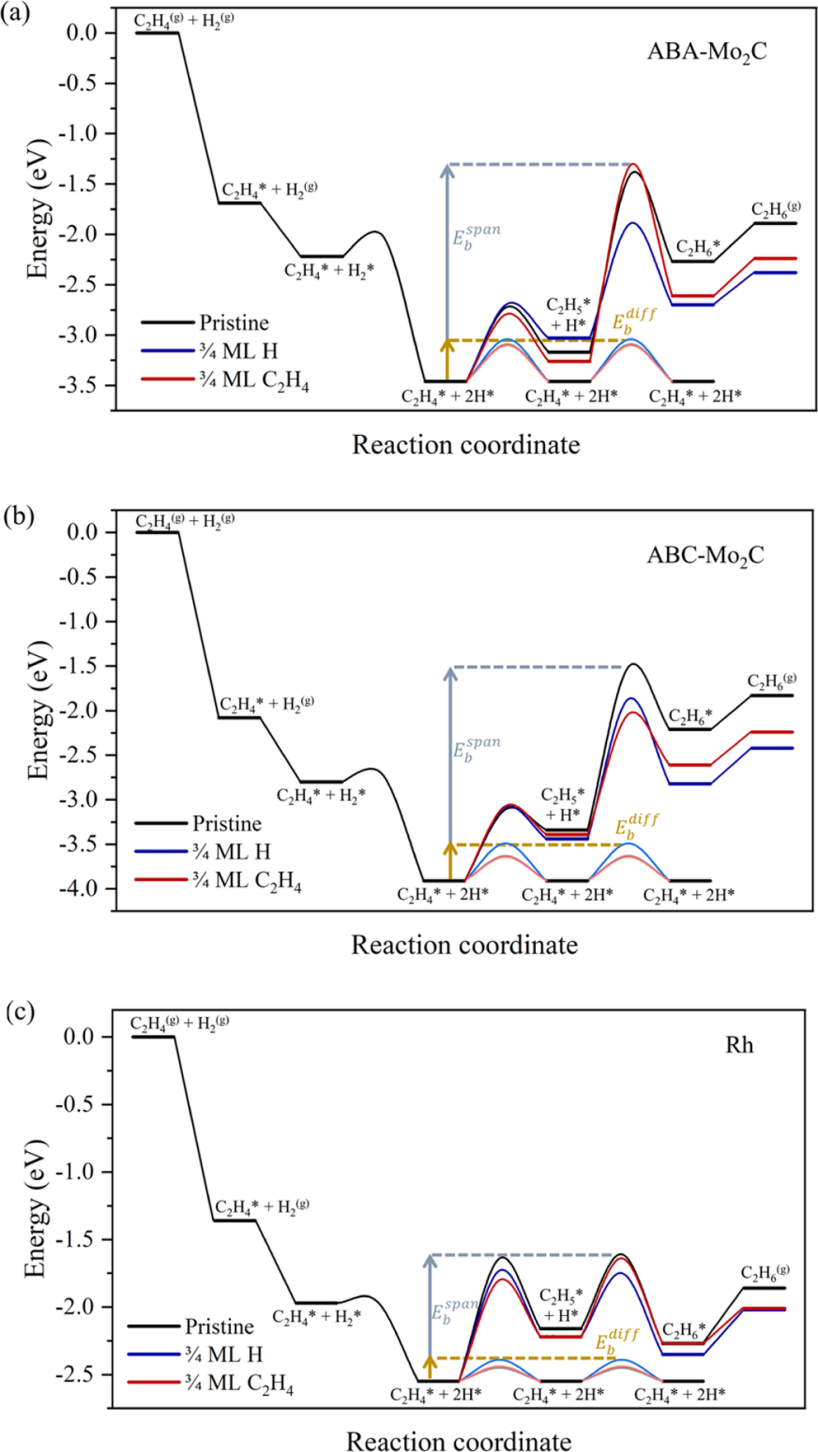
Total reaction energy profiles on the pristine (black) (0001) surfaces of (a) ABA-Mo_2_C and (b) ABC-Mo_2_C, and (c) Rh(111). From C_2_H_4_* + 2H* state, the forward paths are superimposed for 3/4 ML H* (blue), and 3/4 ML C_2_H_4_* (red). The diffusive paths of H* adatoms are shown in lighter shades of the respective colored traces. Note that the pristine diffusive path (gray) and that of the 3/4 ML C_2_H_4_* model (pink) essentially superimpose. All energy values are corrected by the zero energy term (ZPE).

The second hydrogenation step that converts C_2_H_5_* and H* to C_2_H_6_* is slightly exothermic, by −0.11 eV, for Rh(111), with a moderate *E*_b_ of 0.55 eV. However, on the 2D-Mo_2_C models, this step is endothermic, in the range of 0.86–1.32 eV, leading to higher *E*_b_ values varying from 1.77 to 2.11 eV (*cf.*Table S9). Thus, the significant endothermicity of the second H* transfer step on both 2D-Mo_2_C models distinguishes them from the Rh(111) model. The higher energy barrier for the second hydrogenation step on both 2D-Mo_2_C(0001) surfaces is due to the similarly stronger bonding of C_2_H_4_* and C_2_H_5_* species on 2D-Mo_2_C compared to the adsorption energy of C_2_H_6_*, at variance with Rh, where bonding energies are more similar. The similarly high adsorption energies for C_2_H_4_* and C_2_H_5_* species on 2D-Mo_2_C lead to a relatively low barrier for the first hydrogenation step but make the second hydrogenation barrier higher, in agreement with the Brønsted–Evans–Polanyi (BEP) relationships. Overall, the hydrogenation of C_2_H_4_* to C_2_H_6_* is moderately endothermic on Rh(111) by 0.27 eV and has an energy barrier of 0.94 eV according to the span model,^51^ while on ABA- and ABC-Mo_2_C the reaction is endothermic by 1.20 and 1.70 eV, with the span model energy barriers of 2.06 and 2.40 eV, respectively (*cf.*Table S9). These results suggest a more facile hydrogenation of ethene to ethane on Rh(111) relative to both 2D-Mo_2_C models, see Figure 4.

Substrate coverage effects may change the energy barriers, for instance, by lateral interactions. In this context, the high coverage of C_2_H_4_* and H* adatoms under the reaction conditions can be prompted by a stronger ethene adsorption and low barriers for the H_2_ dissociation; see Figures 1, 2, and Table S8, in addition to a higher partial pressure of H_2_ compared to C_2_H_4_. To probe the substrate coverage effects, we considered surface models with 3/4 of a monolayer (ML) of either C_2_H_4_* or H*. Here, coverage is defined based on H* ML with full occupancy of active hollow sites; that is, a H* per 6.9 or 6.4 Å^2^ for 2D-Mo_2_C or Rh, respectively, while assuming C_2_H_4_* occupies a projected area of *ca.* 27.7 Å^2^ on 2D-Mo_2_C, see Figure S2, similar to four sites of H*, a situation copycatted on Rh as well. On the 3/4 ML C_2_H_4_* model, the most stable sampled situation minimized the lateral repulsion between moieties, while in the 3/4 ML H* situation, the simultaneous placement of a C_2_H_4_* moiety left only two empty hollow sites, located near the C_2_H_4_* to allow assessing H_2_ adsorption, dissociation, and C_2_H_4_* hydrogenation steps. Thus, in the 3/4 ML H* model 12 H* adatoms reside on the modeled surface, including the two H* atoms obtained from the H_2_ dissociation. On the 3/4 ML C_2_H_4_* model, there are three C_2_H_4_* molecules, one of which engages in the hydrogenation reaction.

Before addressing ethene hydrogenation at these high coverages, it is worth analyzing H_2_ dissociation at a such higher H* coverage, remembering that, *e.g.*, on Pt(111), the dissociation enthalpy of H_2_ declines at high coverages of H*.^52^ To this end, we removed two vicinal H* adatoms from the 3/4 ML H* model, achieving a ^5^/_8_ ML H* coverage. On that surface, H_2_ adsorption energy slightly decreases to −0.46 and −0.62 eV, and so does the dissociation energy, which decreases to −0.88 and −0.94 eV, with *E*_b_ declining to 0.10 and 0.01 eV, for ABA- and ABC-Mo_2_C, respectively, compared to low-coverage values, see Tables S3 and S8 of the SI. On Rh(111), the *E*_ads_ and dissociation energy reduces to −0.02 and −1.14 eV, as well, with a negligible energy barrier close to 0 eV. Thus, H* coverage seems to reduce the adsorption strength and ease the H_2_ dissociation, yet the effect is much smaller compared to, *e.g.*, the reported data on Pt(111).^52^ With this in mind, the reaction energies, energy barriers, full process energy change, and *E*_b_ values of the span model are listed in Table S10 for the ABA- and ABC-Mo_2_C, and Rh(111) surfaces. The transition states and reaction pathways are presented in Figures S11–S13. These results show that the 3/4 ML coverage of either C_2_H_4_* or H* adatoms has only a minor impact on certain steps (*vide infra*), and so, the full reaction profile is essentially unchanged, see Figure 4, generally unaffected by whether the high coverage situation is found with C_2_H_4_* or H* moieties, also due to the similar surface occupancy of the 3/4 ML C_2_H_4_* or H* models, see above, and expected similar lateral interactions between these surface moieties. This nondependency of activation energies on coverage has been observed, *e.g.*, in allyl alcohol hydrogenation on Rh(111), although for some other hydrogenation reactions, *e.g.*, cyclohexene hydrogenation, the H* coverage was reported to reduce the hydrogenation energy barriers significantly.^53^ On Rh(111), the impact is negligible, making the reaction less endothermic by merely 0.1 eV, and with an *E*_b_ reduced by 0.14 eV in the case of the 3/4 ML H* coverage. The coverage effect on the energy profile of the 2D-Mo_2_C models is similar yet with more pronounced changes; at the 3/4 ML H*-coverage, the span *E*_b_ decreases by 0.49 and 0.35 eV for the ABA-Mo_2_C and ABC-Mo_2_C, respectively. However, for the 3/4 ML C_2_H_4_* coverage, the span *E*_b_ increases by 0.1 eV in ABA-Mo_2_C, yet it decreases by 0.53 eV for ABC-Mo_2_C, a difference attributed to distinct C_2_H_4_* arrangements for the different stackings presented in Figures S11 and S12. Overall, full hydrogenation of C_2_H_4_* is more endothermic, and the reaction barriers are higher on 2D-Mo_2_C compared to Rh(111), regardless of the effects of the stacking or coverages of H* and C_2_H_4_*. The H* adatoms diffusion energy barriers on the 3/4 ML models of C_2_H_4_*, shown in Figure S14, are generally similar to those of the pristine surfaces, with the *E*_b_ variations in the 0.01 eV range for any of the explored Mo_2_C models. Still, for the 3/4 ML coverage of H*, shown in Figure S15, larger diffusion *E*_b_ values of 0.42 eV on ABA- and ABC-Mo_2_C compared to respective values of 0.37 and 0.28 eV on the pristine cases are found, see Table S11.

**Figure 5:**
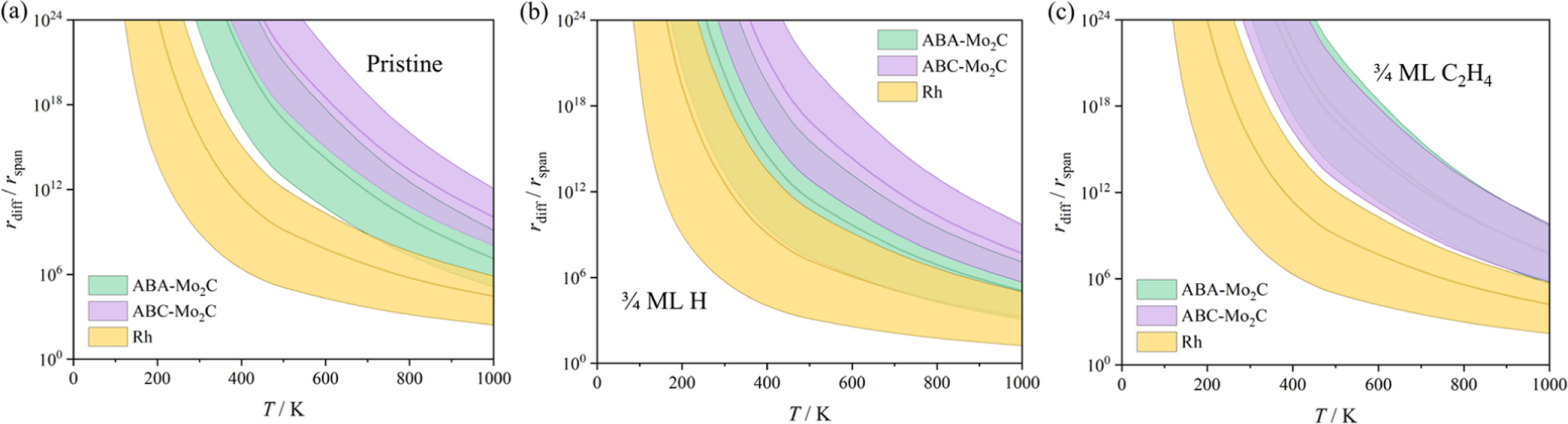
Calculated ratio between the rates of H* diffusion, *r*_diff_, and the reaction rate obtained using the span model energy barrier, *r*_span_, on ABA- and ABC-2D-Mo_2_C (0001) surfaces, and Rh(111) surface, using (a) pristine surfaces, (b) a surface with coverage of 3/4 ML of H* or (c) 3/4 ML of C_2_H_4_*. Shaded regions reflect the DFT uncertainty of ±0.2 eV on the estimated energy barriers.

At this point, one can assess, based on the presented DFT results, the relative fractions of the expected pairwise *vs* nonpairwise hydrogenation pathways, the latter governed by H* diffusion energy barriers. This assessment is based on the estimation of the reaction rates as a function of the working temperature, *T*, *via* the span model energy barriers and the diffusion rates, obtained using transition state theory (TST) and considering 1 bar of reactants. Note that, since the competition of pairwise vs nonpairwise mechanisms relies on reaction vs diffusion rates, microkinetic modeling could provide estimates of reaction rate, although inclusion of diffusion, even if possible, would not affect reaction rate. Thus, as posed, microkinetic modeling would deliver no extra information from estimated rates. Effect of diffusion could be implemented by *ab initio* molecular dynamics, although here one should consider thousands of trajectories, which entails excessive computational costs. A more affordable approach would be kinetic Monte Carlo, although yet with difficulties in tagging spin on H adatom and spin scrambling by diffusion. With this information in hand, one can calculate the ratio between the diffusion rate, *r*_diff_, and the reaction rate obtained using the span model, *r*_span_, *i.e.*, *r*_diff_/*r*_span_. A ratio larger than unity indicates that H* diffusion is faster than the hydrogenation reaction. The obtained *r*_diff_/*r*_span_ ratios for the ABA- and ABC-Mo_2_C(0001) surfaces and that for Rh(111) as a function of temperature are shown in Figure 5.

DFT results show that the nonpairwise mechanism is expected to dominate on any of the model catalytic surfaces studied and that the pairwise mechanism is more likely on Rh(111) than on the 2D-Mo_2_C (0001) surfaces, independent of the H* and C_2_H_4_* coverage of the surfaces. Note, however, that one should account for the DFT accuracy limits of ±0.2 eV. Thus, accuracy limits were added to Figure 5 assuming that the employed PBE-D3 level of calculation is underestimating or overestimating certain *E*_b_ values, in particular, overestimating diffusion barriers *E*_b_ on Rh(111) and the span model *E*_b_ barriers for the hydrogenation reaction on the 2D-Mo_2_C(0001) surfaces, underestimating the diffusion *E*_b_ on the Mo_2_C(0001) surfaces, and hydrogenation span model *E*_b_ barriers on Rh(111). Still, the trends discussed above remain unchanged also after accounting for the accuracy of our DFT approach.

Alternatively, a mechanism based on a concerted addition of H_2_ to C_2_H_4_* that avoids the formation of H* adatoms, if operative, would result in a high selectivity toward pairwise H_2_ addition. Therefore, we have considered also the contribution of an Eley–Rideal mechanism, where the H_2_ molecule reacts with C_2_H_4_* directly from the gas phase, contributing to a pairwise addition via this single-step mechanism. However, all the computational attempts exploring the Eley–Rideal mechanism yielded high DFT energy barriers, *i.e.*, at least 4.84, 5.12, and 2.28 eV for ABA- and ABC-2D-Mo_2_C(0001) and Rh(111) surfaces, respectively, thus larger than the most-demanding energy barrier of the stepwise mechanism (see Table S9 and Figure S16 of the SI). Therefore, a competitive pairwise hydrogenation mechanism that follows the Eley–Rideal kinetics can be discarded.

In what follows, we will discuss experimental results for the estimates of the pairwise and nonpairwise addition pathways obtained in the experiments with parahydrogen addition to propene on Rh/TiO_2_ and Mo_2_C*T*_*x*–500_ catalysts.

### 3.2 Experimental Results

As discussed above, the observation of the NMR signal enhancement (SE) in PHIP experiments requires that the two H atoms of the same p-H_2_ molecule add to an unsaturated bond of a reactant (propene in this case) in a pairwise manner. The SE value is defined by eq 6, with higher SE values corresponding to a higher contribution of pairwise addition pathway to the overall product formation rate. Because the SE is normalized by the amount of product produced in the reaction (see eq 6), the SE values can be directly compared for different conversion levels.

While ethene was used to simplify the DFT calculations described above, PHIP experiments were performed with propene as a substrate. We note that the hydrogenation of ethene with *p*-H_2_, even if entirely pairwise, would not produce observable NMR signal enhancement for the product ethane and thus could not be used to reveal a possible contribution of the pairwise H_2_ addition. This is because the two hydrogen atoms incorporated in ethane upon hydrogenation of ethene are chemically and magnetically equivalent (as in *p*-H_2_), while observation of signal enhancement requires breaking this equivalence in the reaction product. Since the latter condition is satisfied in propane, our hydrogenation experiments use propene. Worthy of note, it is unlikely that the underlying hydrogenation pathways (including the respective adsorption and diffusion properties, *vide supra*) predicted by our DFT calculations for ethene would be significantly different for propene. Therefore, the use of these two homologous olefins is not expected to affect the conclusions of this study.

Mo_2_C*T*_*x*–500_ catalyst was prepared in situ by a 2 h pretreatment of Mo_2_C*T*_*x*_ (100 mg) in the undiluted H_2_ flow at 500 °C, i.e., in conditions that are known to fully reductively defunctionalize the surface termination groups of Mo_2_C*T*_*x*_ (and concurrently generate some C vacancies by removing the carbidic carbon as methane) and provide a 2D-Mo_2_C_1–*x*_ material.^25^ Mo_2_C*T*_*x*–500_ was cooled down to 150 °C after the pretreatment without termination of H_2_ flow and then tested in propene hydrogenation with p-H_2_ using the volume ratio of propene to p-H_2_ of 1:4. Mo_2_C*T*_*x*–500_ showed complete propene conversion at 165 °C when using a flow rate of 26 mL min^–1^; propene conversion decreased to 75% upon increasing the flow rate to 240 mL min^–1^ (Table S12). Noteworthy, propene conversion on Mo_2_C*T*_*x*–500_ declined with increasing temperature, possibly due to the formation of surface carbon deposits. Yet notably low signal enhancements were observed for the reaction product (propane) with Mo_2_C*T*_*x*–500_ across the entire temperature range tested (165–375 °C). More specifically, at temperatures lower than 235 °C, the observed signal enhancements did not exceed 2-fold, indicating an almost entirely nonpairwise H_2_ addition on Mo_2_C*T*_*x*–500_. A slight increase to a (still low yet unambiguously detectable) 10-fold SE with an increase in temperature to 375 °C was observed.

As mentioned above, Rh/TiO_2_ is one of the most efficient catalysts to enable relatively high levels of SE in PHIP experiments at high conversion, *i.e.*, selectivity to the pairwise addition route of up to 8% and a 200-fold SE has been reported previously.^11^ To compare Mo_2_C*T*_*x*–500_ to Rh/TiO_2_ at similar conversions, we mixed 1 wt % Rh/TiO_2_ with a SiC diluent (to help dissipate the heat released of the exothermic propene hydrogenation reaction) and used hydrogenation temperatures in the range of *ca.* 43–150 °C. The obtained propene conversion and SE for Rh/TiO_2_ are presented in Table S12. At low temperatures (*ca.* 43–75 °C), the observed signal enhancements were generally higher for Rh/TiO_2_, with the SE values at ca. 276- to 390-fold. Overall, Mo_2_C*T*_*x*–500_ shows at 150 °C a *ca.* twice higher conversion at the two higher flow rates than Rh/TiO_2_, while SE values differ by *ca.* 2 orders of magnitude (*viz.*, 261–301 for Rh/TiO_2_*vs* 10 for Mo_2_C*T*_*x*–500_).

**Figure 6:**
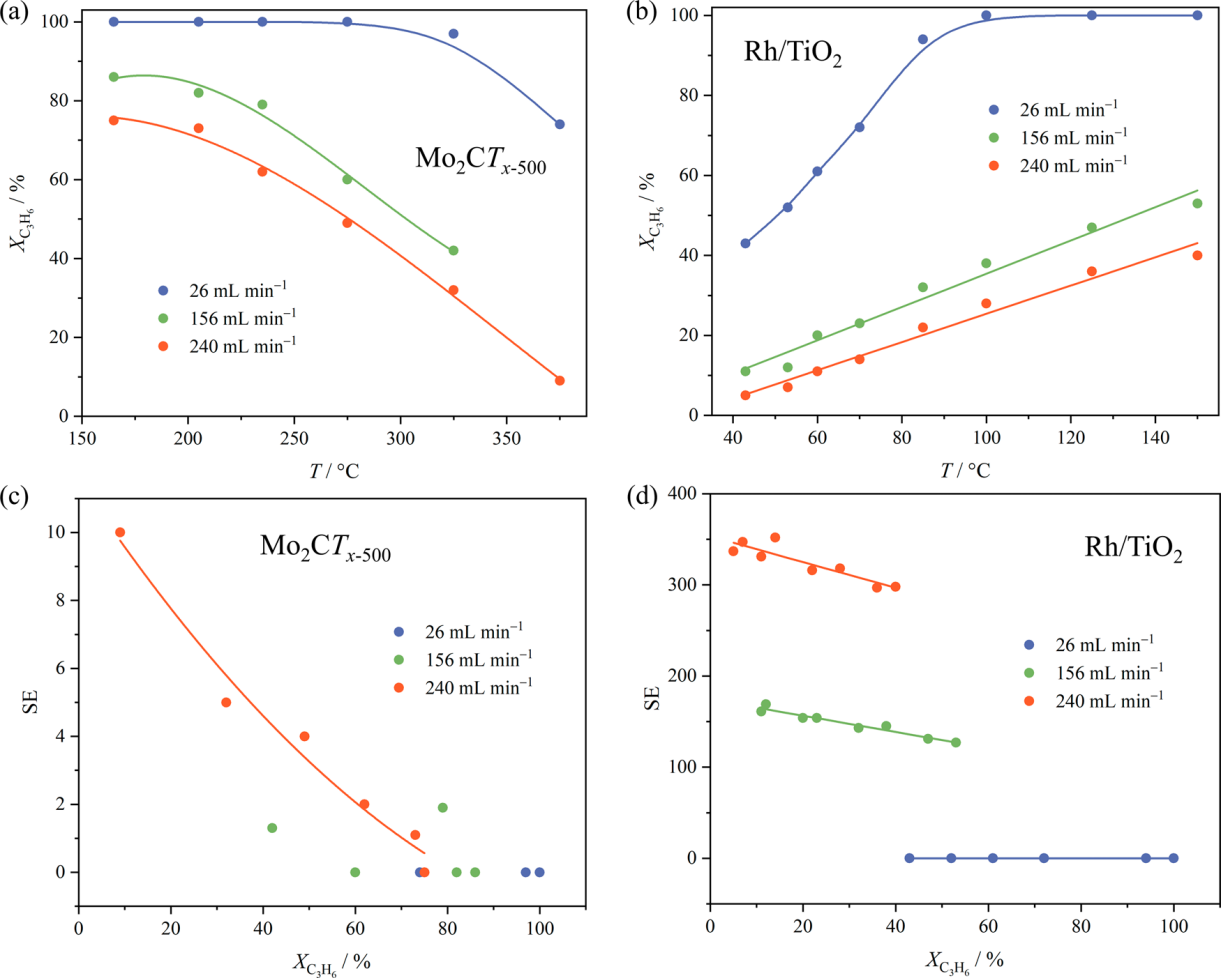
Conversion of propene (X_C_3_H_6__) in its hydrogenation with *p*-H_2_ (1:4 volume ratio) as a function of temperature over (a) Mo_2_C*T*_*x*–500_ and (b) Rh/TiO_2_ catalysts, and (c,d) NMR signal enhancement, SE, as a function of conversion, for the three flow rates used. The lines are added to guide the eye. Note that the metal-based weight-over-flow (W/F) ratios were notably different, i.e., W/F = 2.8, 0.47, 0.30 mg_Mo_ min mL^–1^ and 1.9 × 10^–3^, 3.2 × 10^–4^, 2.1 × 10^–4^ mg_Rh_ min mL^–1^ for the flow rates of 26, 156, and 240 mL min^–1^, respectively.

We note that the observed SE values are systematically larger at higher flow rates. This is due to the relaxation of nuclear spins that drives the nuclear spin system to thermal equilibrium, thereby significantly attenuating the NMR signal enhancement created initially by the pairwise addition of *p*-H_2_. Lower gas flow rates result in longer gas travel time from the reactor to the NMR probe, leading to larger losses of nuclear polarization and lower apparent SE values. To obtain the true SE values, an extrapolation of SE to an infinite flow rate (*i.e.*, zero travel time) is required, yet in practice, this approach may introduce significant uncertainties. Therefore, the SE values obtained at the highest gas flow rate used in the experiments (*i.e.*, 240 mL min^–1^) are taken here as the proxy for the extent of the pairwise hydrogen addition in propene hydrogenation on Mo_2_C*T*_*x*–500_ and Rh/TiO_2_, as presented in Figure 6. At the same time, higher flow rates result in reduced reactant residence time in the reactor and, thus, in lower conversions. For completeness, Table S12 reports both SE and conversion values at all three flow rates used.

### 3.3 Discussion

As shown above, the selectivity to the pairwise hydrogen addition assessed via the experimentally observed NMR signal enhancements is notably larger for the Rh/TiO_2_ relative to Mo_2_C*T*_*x*–500_. Assuming that the hydrogenation proceeds via dissociative chemisorption of H_2_ on a catalyst surface, obtained pairwise selectivities are qualitatively in line with the results of DFT calculations. Indeed, lower ratios between the rates of H* surface diffusion and the hydrogenation reaction (*r*_diff_/*r*_span_) for the Rh(111) compared to those for the 2D-Mo_2_C(0001) surface (representing experimental Rh/TiO_2_ and Mo_2_C*T*_*x*–500_, respectively) imply a higher likelihood for the pairwise addition on Rh(111). However, in quantitative terms, the DFT calculations predict the diffusion to be 4–12 orders of magnitude faster than the hydrogenation reaction under typical experimental conditions. The disparity between the two rates appears too significant to be affected notably by any reasonable fine-tuning of the employed calculational models and the associated computational errors. Furthermore, the calculations additionally suggest that even if an H_2_ molecule would dissociate in the immediate vicinity of an adsorbed ethene molecule, the diffusive separation of H* adatoms is notably faster than their transfer to ethene, and therefore the likelihood of the pairwise hydrogen addition is not increased appreciably.

The observation of PHIP effects on metal surfaces is sometimes ascribed to the presence of adsorbates that lower significantly the diffusive mobility of H* adatoms, favoring thereby the pairwise hydrogen addition.^54^ Indeed, a number of surface sites can be blocked or become blocked if associated with the simultaneous diffusion of H*, both factors slowing down H* diffusion considerably and potentially prompting the pairwise addition. In this context, the present DFT calculations demonstrated no significant changes in the diffusive and reactive rates due to high surface coverages of coadsorbates (3/4 ML of H* or C_2_H_4_*). Within this static picture, the diffusive and reactive channels are not blocked, making the presence of adsorbates an unlikely decisive factor for the emergence of PHIP effects. While the presence of other adsorbates (such as CH_3_CH=CH_2_*) may feature a more pronounced influence on the *r*_diff_/*r*_span_ ratio, the disparity of several orders of magnitude in the *r*_diff_/*r*_span_ ratio observed for the hydrogenation of ethene strongly suggests that adsorbates present at the catalyst surface cannot explain the contribution of the pairwise hydrogenation mechanism, at least for the range of surface coverages addressed in this work.

Alternative explanations of pairwise H_2_ addition thus need to be considered. In particular, the reaction of an H_2_ molecule directly from the gas phase with an adsorbed C_2_H_4_* molecule through an Eley–Rideal mechanism would be expected to result in high pairwise selectivity. However, very high energy barriers revealed by DFT calculations for this mechanism exclude this as a possibility, see Figure S16 of the SI. A few other hydrogenation mechanisms sometimes advanced in catalytic literature generally cannot explain pairwise hydrogenation either because, similar to the Horiuti–Polanyi mechanism, random H* atoms are involved even when the reaction of an alkene or an alkyne with either H_2_^(g)^ or H_2_* (instead of H*) is considered.^7,55,56^ For instance, in the associative mechanism of the partial alkyne hydrogenation, the reaction between adsorbed propyne and H_2_ adds one H atom to propyne but places the second one on the metal surface as H*.^55^ Our DFT results demonstrate that, for the similar configuration C_2_H_5_* + H*, the second H* adatom is much more likely to diffuse away than to complete the hydrogenation cycle, *i.e.*, the underlying substrate-assisted hydrogenation mechanism is also nonpairwise.

Therefore, the quantitative results cannot be reconciled with the theoretically predicted preference for the Horiuti–Polanyi mechanism and other nonpairwise mechanisms of heterogeneous hydrogenations. At the same time, the experimental observations clearly reveal the presence of the pairwise reaction pathway for Rh/TiO_2_ and even for Mo_2_C*T*_*x*–500_ catalysts, for which the calculated *r*_diff_/*r*_span_ ratios are particularly unfavorable. Moreover, this and other experimental studies of PHIP effects demonstrate that pairwise hydrogen addition is essentially omnipresent in hydrogenations catalyzed by various heterogeneous catalysts.^8^ It is thus reasonable to conclude that the results point to the existence of additional reaction route(s) that inherently favor pairwise reaction pathway, operating concurrently with the dominant Horiuti–Polanyi mechanism and contributing measurably to the overall reaction yield. When such a concurrent mechanism involves the dissociation of H_2_, the migration (and therefore, the randomization) of the formed H* species should be strongly suppressed, as is, for instance, the case for molecular transition metal catalysts that operate via the oxidative addition of H_2_, olefin insertion, and reductive elimination steps. In the case of Rh/TiO_2_, the presence of such “pairwise-selective” sites could result from an strong metal-support interaction (SMSI) effect. When SMSI effects are not available, a plausible mechanism could rely on a blocked diffusion of H* adatoms arising from occupied sites and the simultaneous competing diffusion of many H* adatoms, dynamically preventing diffusion until a nearby site is freed, an aspect not considered in the computation of diffusion rates. This would imply that H* adatoms formed after H_2_ adsorption and dissociation nearby C_2_H_4_* cannot diffuse away as fast as predicted due to the dynamic site blocking, particularly for coverages higher than the explored 0.75 ML coverage. In such a situation, H* would have fewer chances to diffuse, and concomitantly, both generated H* atoms from p-H_2_ would be more likely added in a pairwise manner to C_2_H_4_*.^57^ Alternatively, a mechanism based on a concerted addition of H_2_* to a substrate that completely avoids the formation of H* adatoms and features competitive reaction barriers relative to the Horiuti–Polanyi pathway would result in a high selectivity toward pairwise H_2_ addition in the hydrogenation of unsaturated hydrocarbons. Future studies should explore the possibilities outlined above.

## 4 Conclusions

Here, first-principles DFT calculations were used to elucidate a more detailed reaction mechanism of the ethene hydrogenation on the well-defined model Rh(111) surface and 2D-Mo_2_C(0001) with ABC- and ABA-stacking. Consistent results from both DFT calculations and experimental observations indicate that these catalysts are capable of effectively catalyzing the hydrogenation of ethene, which aligns with predictions based on adsorption rates that decrease sequentially for C_2_H_4_, H_2_, and C_2_H_6_. Furthermore, the DFT results show that although 2D-Mo_2_C and Rh(111) surfaces adsorb H_2_, dissociate H_2_, and diffuse H* adatoms with comparable barriers, regardless of the surface coverage with H* and C_2_H_4_* adsorbates in a model ethene hydrogenation reaction, the H* transfer steps for the hydrogenation of C_2_H_5_* to C_2_H_6_* are distinct. Specifically, while the first hydrogen transfer, to form C_2_H_5_* from C_2_H_4_* and H*, proceeds with similar barriers on Rh(111) and 2D-Mo_2_C, the hydrogenation of ethyl species to ethane is endothermic on 2D-Mo_2_C models and features significantly higher energy barriers than on Rh(111). This leads to a lower predicted rate of the ethene hydrogenation reaction on 2D-Mo_2_C (in agreement with the experiment).

The DFT calculations were combined, for the first time, with experimental studies of hydrogenation with parahydrogen to address the origin of the nonpairwise vs pairwise H_2_ addition. The hydrogenation of propene on 2D-Mo_2_C_1–*x*_ and Rh/TiO_2_ catalysts at comparable conversions resulted in an appreciable enhancement of the NMR signals of the reaction product (propane), which indicates unambiguously that the pairwise addition of H_2_ to propene contributes measurably to the reaction rate, in line also with the previous studies that relied on parahydrogen to demonstrate the existence of the pairwise hydrogenation pathway on various surfaces. Importantly, our DFT study highlighted that diffusive migration of H* adatoms on a catalyst surface, which is an essential part of the Horiuti–Polanyi hydrogenation mechanism, is notably faster relative to the rate of H* addition to ethene, such that only randomized H* adatoms are added to the alkene. This inference is not altered measurably when considering significantly high catalyst surface coverages and other reaction conditions, implying no adsorbate lateral interaction hindrances.

While the experimentally established preference for the pairwise mechanism on Rh relative to 2D-Mo_2_C is qualitatively explained based on the inherent H* diffusion differences between these catalysts, in more quantitative terms, the pairwise H_2_ addition, with both H atoms of the same H_2_ molecule ending up in the same product molecule, is predicted to be markedly less probable on any studied catalyst. Therefore, this combined theoretical and experimental study clearly demonstrates the predominance of the widely accepted Horiuti–Polanyi hydrogenation mechanism, which cannot explain the measurable contribution of the pairwise hydrogenation pathway as experimentally observed. Alternative reaction pathways, such as the identified concerted transition states for the H_2_ addition following the Eley–Rideal pathway, were ruled out based on their high reaction barriers. One remaining plausible explanation is the diffusion hindrance of H* adatoms due to a dynamic surface site blocking at coverages higher than the currently explored 0.75 ML of H* or C_2_H_4_*, potentially prompting the pairwise addition. However, this remains to be confirmed in subsequent studies. Overall, the underlying pairwise hydrogenation mechanism avoids the randomization of hydrogen atoms either by preventing diffusion and scrambling of H* adatoms or by excluding entirely the involvement of H* adatoms in the reaction pathway.

## Supporting Information Available

Configurations of catalysts, adsorbates, and the lowest-energy surface sites; Gibbs free/total adsorption energies of adsorbates; estimations of rates; reaction energies, energy barriers, and atomic structure view for H diffusion, H_2_ dissociation and reaction process; conversion of propene, X_C_3_H_6__, and NMR signal enhancement of propane, SE (PDF)

## References

[ref1] Zhang L.; Zhou M.; Wang A.; Zhang T. Selective Hydrogenation over Supported Metal Catalysts: From Nanoparticles to Single Atoms. Chem. Rev. 2020, 120, 683–733. 10.1021/acs.chemrev.9b00230. 31549814

[ref2] Aireddy D. R.; Ding K. Heterolytic Dissociation of H_2_ in Heterogeneous Catalysis. ACS Catal. 2022, 12, 4707–4723. 10.1021/acscatal.2c00584.

[ref3] Thomas S. P.; Greenhalgh M. D. Heterogeneous Hydrogenation of C=C and C≡C Bonds. In Comprehensive Organic Synthesis, Vol. 1; Knochel P., Molander J. A., Eds. Elsevier: Amsterdam, 2014; pp 564−604.

[ref4] Mattson B.; Foster W.; Greimann J.; Hoette T.; Le N.; Mirich A.; Wankum S.; Cabri A.; Reichenbacher C.; Schwanke E. Heterogeneous Catalysis: The Horiuti–Polanyi Mechanism and Alkene Hydrogenation. J. Chem. Educ. 2013, 90, 613–619. 10.1021/ed300437k.

[ref5] Horiuti J.; Polanyi M. A Catalysed Reaction of Hydrogen with Water. Nature 1933, 132, 819 10.1038/132819a0.

[ref6] Horiuti I.; Polanyi M. Exchange Reactions of Hydrogen on Metallic Catalysts. Trans. Faraday Soc. 1934, 30, 1164–1172. 10.1039/tf9343001164.

[ref7] Yang B.; Burch R.; Hardacre C.; Hu P.; Hughes P. Selective Hydrogenation of Acetylene over Cu(211), Ag(211) and Au(211): Horiuti–Polanyi Mechanism vs. Non-Horiuti–Polanyi Mechanism. Catal. Sci. Technol. 2017, 7, 1508–1514. 10.1039/C6CY02587K.

[ref8] Pokochueva E. V.; Burueva D. B.; Salnikov O. G.; Koptyug I. V. Heterogeneous Catalysis and Parahydrogen-Induced Polarization. ChemPhysChem 2021, 22, 1421–1440. 10.1002/cphc.202100153. 33969590

[ref9] Kovtunov K. V.; Beck I. E.; Bukhtiyarov V. I.; Koptyug I. V. Observation of Parahydrogen-Induced Polarization in Heterogeneous Hydrogenation on Supported Metal Catalysts. Angew. Chem., Int. Ed. 2008, 47, 1492–1495. 10.1002/anie.200704881. 18205153

[ref10] Salnikov O. G.; Liu H. J.; Fedorov A.; Burueva D. B.; Kovtunov K. V.; Copéret C.; Koptyug I. V. Pairwise Hydrogen Addition in the Selective Semihydrogenation of Alkynes on Silica-Supported Cu Catalysts. Chem. Sci. 2017, 8, 2426–2430. 10.1039/C6SC05276B. 28451349 PMC5369404

[ref11] Pokochueva E. V.; Burueva D. B.; Kovtunova L. M.; Bukhtiyarov A. V.; Gladky A. Y.; Kovtunov K. V.; Koptyug I. V.; Bukhtiyarov V. I. Mechanistic In Situ Investigation of Heterogeneous Hydrogenation over Rh/TiO_2_ Catalysts: Selectivity, Pairwise Route and Catalyst Nature. Faraday Discuss. 2021, 229, 161–175. 10.1039/C9FD00138G. 33720219

[ref12] Kovtunov K. V.; Barskiy D. A.; Salnikov O. G.; Burueva D. B.; Khudorozhkov A. K.; Bukhtiyarov A. V.; Prosvirin I. P.; Gerasimov E. Y.; Bukhtiyarov V. I.; Koptyug I. V. Strong Metal–Support Interactions for Palladium Supported on TiO_2_ Catalysts in the Heterogeneous Hydrogenation with Parahydrogen. ChemCatChem 2015, 7, 2581–2584. 10.1002/cctc.201500618.

[ref13] Zhou R.; Zhao E. W.; Cheng W.; Neal L. M.; Zheng H.; Quiñones R. E.; Hagelin-Weaver H. E.; Bowers C. R. Parahydrogen-Induced Polarization by Pairwise Replacement Catalysis on Pt and Ir Nanoparticles. J. Am. Chem. Soc. 2015, 137, 1938–1946. 10.1021/ja511476n. 25629434

[ref14] Salnikov O. G.; Burueva D. B.; Gerasimov E. Y.; Bukhtiyarov A. V.; Khudorozhkov A. K.; Prosvirin I. P.; Kovtunova L. M.; Barskiy D. A.; Bukhtiyarov V. I.; Kovtunov K. V.; Koptyug I. V. The Effect of Oxidative and Reductive Treatments of Titania-Supported Metal Catalysts on the Pairwise Hydrogen Addition to Unsaturated Hydrocarbons. Catal. Today 2017, 283, 82–88. 10.1016/j.cattod.2016.02.030.

[ref15] Zhao E. W.; Zheng H.; Ludden K.; Xin Y.; Hagelin-Weaver H. E.; Bowers C. R. Strong Metal–Support Interactions Enhance the Pairwise Selectivity of Parahydrogen Addition over Ir/TiO_2_. ACS Catal. 2016, 6, 974–978. 10.1021/acscatal.5b02632.

[ref16] Zhivonitko V. V.; Skovpin I. V.; Szeto K. C.; Taoufik M.; Koptyug I. V. Parahydrogen-Induced Polarization Study of the Silica-Supported Vanadium Oxo Organometallic Catalyst. J. Phys. Chem. C 2018, 122, 4891–4900. 10.1021/acs.jpcc.7b12069. PMC6150668 30258526

[ref17] Parastaev A.; Muravev V.; Osta E. H.; Kimpel T. F.; Simons J. F. M.; van Hoof A. J. F.; Uslamin E.; Zhang L.; Struijs J. J. C.; Burueva D. B.; Pokochueva E. V.; Kovtunov K. V.; Koptyug I. V.; Villar-Garcia I. J.; Escudero C.; Altantzis T.; Liu P.; Béché A.; Bals S.; Kosinov N.; Hensen J. M. Breaking Structure Sensitivity in CO_2_ Hydrogenation by Tuning Metal–Oxide Interfaces in Supported Cobalt Nanoparticles. Nat. Catal. 2022, 5, 1051–1060. 10.1038/s41929-022-00874-4.

[ref18] Burueva D. B.; Smirnov A. A.; Bulavchenko O. A.; Prosvirin I. P.; Gerasimov E. Y.; Yakovlev V. A.; Kovtunov K. V.; Koptyug I. V. Pairwise Parahydrogen Addition Over Molybdenum Carbide Catalysts. Top. Catal. 2020, 63, 2–11. 10.1007/s11244-019-01211-z.

[ref19] Boudart M.; Djega-Mariadassou G. Kinetics of Heterogeneous Catalytic Reactions. Princeton University Press, 1984.

[ref20] Farkas A. Part. II.—Catalytic Reactions of Hydrocarbons. The Activation of Hydrogen in Catalytic Reactions of Hydrocarbons. Trans. Faraday Soc. 1939, 35, 906–917. 10.1039/TF9393500906.

[ref21] Baráth E. Hydrogen Transfer Reactions of Carbonyls, Alkynes, and Alkenes with Noble Metals in the Presence of Alcohols/Ethers and Amines as Hydrogen Donors. Catalysts 2018, 8, 671 10.3390/catal8120671.

[ref22] Pokochueva E. V.; Kountoupi E.; Janák M.; Kuznetsov D. A.; Prosvirin I. P.; Müller C.; Fedorov A.; Koptyug I. V. Implications for the Hydrogenation of Propyne and Propene with Parahydrogen due to the *in situ* Transformation of Rh_2_C to Rh^0^/C. ChemPhysChem 2024, e202400270 10.1002/cphc.202400270. 38837531

[ref23] Anasori B.; Lukatskaya M. R.; Gogotsi Y. 2D Metal Carbides and Nitrides (MXenes) for Energy Storage. Nat. Rev. Mater. 2017, 2, 16098 10.1038/natrevmats.2016.98.

[ref24] Zhou H.; Chen Z.; Kountoupi E.; Tsoukalou A.; Abdala P. M.; Florian P.; Fedorov A.; Müller C. R. Two-Dimensional Molybdenum Carbide 2D-Mo_2_C as a Superior Catalyst for CO_2_ Hydrogenation. Nat. Commun. 2021, 12, 5510 10.1038/s41467-021-25784-0. 34535647 PMC8448824

[ref25] Kountoupi E.; Barrios A. J.; Chen Z.; Müller C. R.; Ordomsky V. V.; Comas-Vives A.; Fedorov A. The Impact of Oxygen Surface Coverage and Carbidic Carbon on the Activity and Selectivity of Two-Dimensional Molybdenum Carbide (2D-Mo_2_C) in Fischer–Tropsch Synthesis. ACS Catal. 2024, 14, 1834–1845. 10.1021/acscatal.3c03956. 38327645 PMC10845113

[ref26] Yan Y.; Sall D.; Loupias L.; Célérier S.; Aouine M.; Bargiela P.; Prévot M.; Morfin F.; Piccolo L. MXene-Supported Single-Atom and Nano Catalysts for Effective Gas-Phase Hydrogenation Reactions. Mater. Today Catal. 2023, 2, 100010 10.1016/j.mtcata.2023.100010.

[ref27] Koptyug I. V.; Kovtunov K. V.; Burt S. R.; Anwar M. S.; Hilty C.; Han S. I.; Pines A.; Sagdeev R. Z. *para*-Hydrogen-Induced Polarization in Heterogeneous Hydrogenation Reactions. J. Am. Chem. Soc. 2007, 129, 5580–5586. 10.1021/ja068653o. 17408268

[ref28] Wade L. G. Organic Chemistry, 6th ed.; Pearson Prentice Hall, 2006; p 279.

[ref29] Mao Z.; Xie Z.; Chen J. G. Comparison of Heterogeneous Hydroformylation of Ethylene and Propylene over RhCo_3_/MCM-41 Catalysts. ACS Catal. 2021, 11, 14575–14585. 10.1021/acscatal.1c04359.

[ref30] Liu R. Ideal Site Geometry for Heterogeneous Catalytic Reactions: A DFT Study. Catalysts 2024, 14, 34 10.3390/catal14010034.

[ref31] Kresse G.; Furthmüller J. Efficient Iterative Schemes for Ab Initio Total-Energy Calculations Using a Plane-Wave Basis Set. Phys. Rev. B 1996, 54, 11169 10.1103/PhysRevB.54.11169. 9984901

[ref32] Perdew J. P.; Burke K.; Ernzerhof M. Generalized Gradient Approximation Made Simple. Phys. Rev. Lett. 1996, 77, 3865 10.1103/PhysRevLett.77.3865. 10062328

[ref33] Grimme S.; Antony J.; Ehrlich S.; Krieg H. A Consistent and Accurate Ab Initio Parametrization of Density Functional Dispersion Correction (DFT-D) for the 94 Elements H-Pu. J. Chem. Phys. 2010, 132, 154104 10.1063/1.3382344. 20423165

[ref34] Blöchl P. E. Projector Augmented-Wave Method. Phys. Rev. B 1994, 50, 17953 10.1103/PhysRevB.50.17953. 9976227

[ref35] Kresse G.; Joubert D. From Ultrasoft Pseudopotentials to the Projector Augmented-Wave Method. Phys. Rev. B 1999, 59, 1758 10.1103/PhysRevB.59.1758.

[ref36] Vega L.; Ruvireta J.; Viñes F.; Illas F. Jacob’s Ladder as Sketched by Escher: Assessing the Performance of Broadly Used Density Functionals on Transition Metal Surface Properties. J. Chem. Theory Comput. 2018, 14, 395–403. 10.1021/acs.jctc.7b01047. 29182868

[ref37] Gouveia J. D.; Viñes F.; Illas F.; Gomes J. R. B. MXenes Atomic Layer Stacking Phase Transitions and Their Chemical Activity Consequences. Phys. Rev. Mater. 2020, 4, 054003 10.1103/PhysRevMaterials.4.054003.

[ref38] Monkhorst H. J.; Pack J. D. Special Points for Brillouin-Zone Integrations. Phys. Rev. B 1976, 13, 5188 10.1103/PhysRevB.13.5188.

[ref39] Zhang Y.; Kitchaev D. A.; Yang J.; Chen T.; Dacek S. T.; Sarmiento-Pérez R. A.; Marques M. A. L.; Peng H.; Ceder G.; Perdew J. P.; Sun J. Efficient First-Principles Prediction of Solid Stability: Towards Chemical Accuracy. npj Comput. Mater. 2018, 4, 9 10.1038/s41524-018-0065-z.

[ref40] Li Q.; Ouyang Y.; Lu S.; Bai X.; Zhang Y.; Shi L.; Ling C.; Wang J. Perspective on Theoretical Methods and Modeling Relating to Electro-Catalysis Processes. Chem. Commun. 2020, 56, 9937–9949. 10.1039/D0CC02998J. 32644088

[ref41] Cho J.; Lim T.; Kim H.; Meng L.; Kim J.; Lee S.; Lee J. H.; Jung G. Y.; Lee K. S.; Viñes F.; Illas F.; Exner K. S.; Joo S. H.; Choi C. H. Importance of Broken Geometric Symmetry of Single-Atom Pt Sites for Efficient Electrocatalysis. Nat. Commun. 2023, 14, 3233 10.1038/s41467-023-38964-x. 37270530 PMC10239452

[ref42] Gouveia J. D.; Morales-García Á.; Viñes F.; Illas F.; Gomes J. R. B. MXenes as Promising Catalysts for Water Dissociation. Appl. Catal., B 2020, 260, 118191 10.1016/j.apcatb.2019.118191.

[ref43] Morales-Salvador R.; Gouveia J. D.; Morales-García Á.; Viñes F.; Gomes J. R. B.; Illas F. Carbon Capture and Usage by MXenes. ACS Catal. 2021, 11, 11248–11255. 10.1021/acscatal.1c02663.

[ref44] Henkelman G.; Jónsson H. A Dimer Method for Finding Saddle Points on High Dimensional Potential Surfaces Using only First Derivatives. J. Chem. Phys. 1999, 111, 7010–7022. 10.1063/1.480097.

[ref45] Reuter K.; Scheffler M. Composition, Structure, and Stability of RuO_2_(110) as a Function of Oxygen Pressure. Phys. Rev. B 2001, 65, 035406 10.1103/PhysRevB.65.035406.

[ref46] Halim J.; Kota S.; Lukatskaya M. R.; Naguib M.; Zhao M. Q.; Moon E. J.; Pitock J.; Nanda J.; May S. J.; Gogotsi Y.; Barsoum M. W. Synthesis and Characterization of 2D Molybdenum Carbide (MXene). Adv. Funct. Mater. 2016, 26, 3118–3127. 10.1002/adfm.201505328.

[ref47] Deeva E. B.; Kurlov A.; Abdala P. M.; Lebedev D.; Kim S. M.; Gordon C. P.; Tsoukalou A.; Fedorov A.; Müller C. R. In Situ XANES/XRD Study of the Structural Stability of Two-Dimensional Molybdenum Carbide Mo_2_CT_*x*_: Implications for the Catalytic Activity in the Water–Gas Shift Reaction. Chem. Mater. 2019, 31, 4505–4513. 10.1021/acs.chemmater.9b01105.

[ref48] Jurado A.; Morales-García Á.; Viñes F.; Illas F. Molecular Mechanism and Microkinetic Analysis of the Reverse Water Gas Shift Reaction Heterogeneously Catalyzed by the Mo_2_C MXene. ACS Catal. 2022, 12, 15658–15667. 10.1021/acscatal.2c04489.

[ref49] Bond G. C. Metal-Catalysed Reactions of Hydrocarbons; Springer: Boston, MA, USA, 2005.

[ref50] Pozzo M.; Alfè D.; Amieiro A.; French S.; Pratt A. Hydrogen Dissociation and Diffusion on Ni- and Ti-Doped Mg(0001) Surfaces. J. Chem. Phys. 2008, 128, 094703 10.1063/1.2835541. 18331106

[ref51] Kozuch S.; Shaik S. How to Conceptualize Catalytic Cycles? The Energetic Span Model. Acc. Chem. Res. 2011, 44, 101–110. 10.1021/ar1000956. 21067215

[ref52] Podkolzin S. G.; Watwe R. M.; Yan Q.; de Pablo J. J.; Dumesic J. A. DFT Calculations and Monte Carlo Simulations of the Co-Adsorption of Hydrogen Atoms and Ethylidyne Species on Pt(111). J. Phys. Chem. B 2001, 105, 8550–8562. 10.1021/jp0104076.

[ref53] Li H.; Ding Z. Hydrogen Coverage Dependent C=C Hydrogenation Activity on Rh(111). Chem. Phys. Lett. 2020, 746, 137287 10.1016/j.cplett.2020.137287.

[ref54] McCormick J.; Korchak S.; Mamone S.; Ertas Y.; Liu Z.; Verlinsky L.; Wagner S.; Gloggler S.; Bouchard L. Over 12% Polarization and 20 minute Lifetime of ^15^N on Choline Derivative Utilizing Parahydrogen and Rh Nanocatalyst in Water. Angew. Chem., Int. Ed. 2018, 57, 10692–10696. 10.1002/anie.201804185. 29923285

[ref55] Vilé G.; Baudouin D.; Remediakis I. N.; Copéret C.; López N.; Pérez-Ramírez J. Silver Nanoparticles for Olefin Production: New Insights into the Mechanistic Description of Propyne Hydrogenation. ChemCatChem 2013, 5, 3750–3759. 10.1002/cctc.201300569.

[ref56] Yang B.; Gong X.-Q.; Wang H.-F.; Cao X.-M.; Rooney J. J.; Hu P. Evidence To Challenge the Universality of the Horiuti–Polanyi Mechanism for Hydrogenation in Heterogeneous Catalysis: Origin and Trend of the Preference of a Non-Horiuti–Polanyi Mechanism. J. Am. Chem. Soc. 2013, 135, 15244–15250. 10.1021/ja408314k. 24032528

[ref57] Dong Y.; Ebrahimi M.; Tillekaratne A.; Zaera F. Direct Addition Mechanism during the Catalytic Hydrogenation of Olefins over Platinum Surfaces. J. Phys. Chem. Lett. 2016, 7, 2439–2443. 10.1021/acs.jpclett.6b01103. 27309969

