# Supplementary material for: Contrasting Metallic (Rh0) and Carbidic (2D-Mo2C MXene) Surfaces in Olefin Hydrogenation Provides Insights on the Origin of the Pairwise Hydrogen Addition
Source: ACS Catal. 2024 Aug 6;14(16):12500–11. doi: 10.1021/acscatal.4c02534 (PMC11334177; doi:10.1021/acscatal.4c02534)
Supplement: Supplementary file 1 — cs4c02534_si_001.pdf [file cs4c02534_si_001.pdf]

Contrasting Metallic (Rh^0) and Carbidic (2D-Mo_2C MXene) Surfaces in Olefin Hydrogenation Provides Insights on the Origin of the Pairwise Hydrogen Addition

Ling Meng,[1] Ekaterina V. Pokochueva,[2,+] Zixuan Chen,[3] Alexey Fedorov,[3,*] Francesc Viñes,[1,*] Francesc Illas,[1] Igor V. Koptyug[2]

[1]*Departament de Ciència de Materials i Química Física & Institut de Química Teòrica i Computacional (IQTCUB), Universitat de Barcelona, c/ Martí i Franquès 1-11, 08028 Barcelona, Spain.*

[2]*International Tomography Center SB RAS, 3A Institutskaya St., Novosibirsk, 630090, Russian Federation.*

[3]*Department of Mechanical and Process Engineering, ETH Zürich, Leonhardstrasse 21, Zürich, 8092, Switzerland.*

[+]*Current address: Université Claude Bernard Lyon 1, CRMN UMR-5082, CNRS, ENS Lyon, Villeurbanne 69100 France.*

*Corresponding authors: Alexey Fedorov (fedoroal@ethz.ch), Francesc Viñes (francesc.vines@ub.edu).

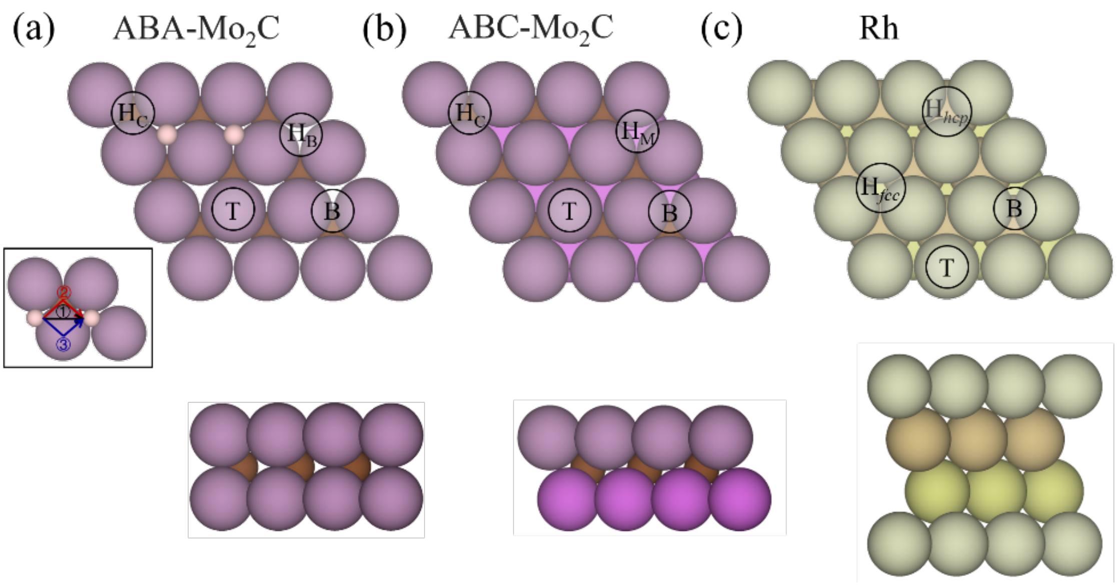

Figure S1. Top (upper images) and side (lower images) views of *p*(4×4) MXene-derived 2D-Mo_2C (0001) surface with (a) ABA stacking and (b) ABC stacking, as well as (c) Rh (111) surface. High symmetry sites are specified, including top (T) and bridge (B) sites for all surface models, hollow carbon (H_C), hollow blank (H_B), and hollow metal (H_M) for ABA- and ABC-stacked 2D-Mo_2C (0001), and hexagonal close-packed hollow (H_{hcp}) and face-centred cubic hollow (H_{fcc}) on Rh (111). C and H atoms are represented by brown and white spheres, respectively, while Mo and Rh atoms are shown as pinkish and greenish spheres, with different levels of shading depending on their stacking position. The red, black and blue lines represent the three modes of H adatom diffusion explored departing from any competitive hollow site minimum.

Section S1: Gibbs Free Energies of Adsorption

The reaction species Gibbs free energies of adsorption, ΔG^{ad}, as a function of the working temperature, T, and the species partial pressure, p, are approximated as:

$$\Delta G^{ad}(T,\ p) \approx\ [E^{total}(N_i, N_M) +\ E^{ZPE}_{(N_i,N_M)}] - E^{total}(0, N_M) - N_i[E^{total}_i\ +\ E^{ZPE}_i]\ - N_i\Delta\mu_i(T,p) \quad \text{(S1)},$$

where N_i denotes the count of adsorbed species, normally one in the studied system, while E^{total}_i, $E^{ZPE}_{(N_i,N_M)}$ and E^{ZPE}_i represent the total energy, and the zero point energy (ZPE) contributions of the adsorbed species, and that species in vacuum, respectively. N_M, for instance, would be the number of metal atoms, *e.g.* in the Rh (111) slab model, while for the Mo_2C models, this would be the number of each of substrate atom types. Since the number of substrate atoms is invariant in our study, $E^{total}(N_i, N_M) =\ E_{i/sub}$, and $E^{total}(0, N_M) =\ E_{sub}$, where E_{sub} and $E_{i/sub}$ are the total energies of the pristine surface model and of the surface model with the ith species adsorbed upon, respectively. Aside, $\Delta\mu_i(T,p)$ is the chemical potential change of the adsorbed species with respect to the gas phase, where details on how to estimate it are provided below. E^{ZPE}_i can be obtained from vibrational frequencies, *viz.*:

$$E^{ZPE}_i\ = \frac{1}{2}\sum_{n=1}^{NMV} h\nu_n \quad \text{(S2)},$$

where h is the Planck's constant, and ν_n is the vibrational frequency for each normal mode n of vibration (NMV), *i.e.*, $3N$−5 for linear molecules in vacuum, $3N$−6 for non-linear molecules in vacuum, and $3N$ for adsorbed atoms/molecules, where N is the number of atoms (taking into account the loss of free translations and rotations that are effectively converted into vibrations upon adsorption). For C_2H_4, C_2H_6, and H_2 molecules, the gas reference is well-defined. For radical species such as C_2H_5 and H adatoms it is convenient to express its chemical potential as a combination of gas phase species, *i.e.*, $\mu_H(T,p)\ =\ 1/2\ \mu_{H_2}(T,p)$, and $\mu_{C_2H_5}(T,p)\ = \mu_{C_2H_4}(T,p) +\ 1/2\ \mu_{H_2}(T,p)$. Consequently, one can use *ab initio* thermodynamics (AIT) to express the chemical potential variations $\Delta\mu_i(T,p)$ as a product containing kinetic, rotational, vibrational, and electronic contributions according to:

$$\Delta\mu_i(T,p) =\ k_BT\left\{ln\left[\left(\frac{2\pi m_i}{h^2}\right)^{3/2}\frac{(k_BT)^{5/2}}{p_i}\right] +\ ln\left(\frac{k_BT}{\sigma_i^{sym}B_{0,i}}\right) -\ \sum_{n=1}^{NMV} ln\left[1 - exp\left(\frac{-h\nu_{n,i}}{k_BT}\right)\right] + ln\left(I_i^{spin}\right)\right\} \quad \text{(S3)},$$

where k_B is the Boltzmann's constant, m_i the mass of the ith molecule, p_i denotes the partial pressure of the ith species, σ_i^{sym} is the symmetry number of ith molecule —2 for H_2, 4 for C_2H_4, and 6 for C_2H_6,[1] $B_{0,i}$ is the rotational constant, computed as $B_{0,i} = \frac{\hbar^2}{2I_i}$, where I_i corresponds to the moment of inertia of the molecule, given by $I_i = \sum_a m_a r_a{}^2$, where m_a is the mass of the atoms composing the ith molecule and r_a refers to the distance of the a atom centre to the molecular centre of mass. Moreover, each of the vibrational normal modes of the ith molecule is assigned by $\nu_{n,i}$, while I_i^{spin} is the electronic spin degeneracy of the ground state. Since all the molecules considered here feature a singlet ground state, this term was neglected in the present study.

Section S2: Estimations of Rates

The non-activated adsorption rate of a species ($r_{\rm ads}$) can be gained using collision theory,[2] as;

$$r_{ads} = \frac{S_0 \cdot p_i \cdot A}{\sqrt{2\pi \cdot m_i \cdot k_B \cdot T}} \quad \text{(S4)},$$

where S_0 is the initial sticking coefficient, $p_{\rm i}$ the partial pressure of H_2, C_2H_4, or C_2H_6 in the gas phase, and A represents the surface area of an adsorption site, estimated by dividing the surface supercell area, see Figure S1, by the number of possible adsorption sites.

The desorption rate, $r_{\rm des}$, is estimated from the transition state theory (TST) and assuming that the desorbed TS is a late two-dimensional (2D) transition state,[3] where the energy barrier is the desorption energy, $\Delta E^{i}_{\rm des}$, which is simply a negative of the adsorption energy, $\Delta E^{i}_{\rm ads}$, see main text. Thus, *viz.*:

$$r_{des} = v_{des} \cdot exp\left(\frac{\Delta E^{i}_{\rm ads}}{k_B \cdot T}\right);\; v_{des} = \frac{k_B \cdot T}{h} \frac{q^{gas}_{trans,2D} \cdot q^{gas}_{rot} \cdot q^{gas}_{vib}}{q^{ads}_{vib}} \quad \text{(S5)},$$

where $\Delta E^{i}_{\rm ads}$ is, here, non-ZPE corrected. Note that in such rates definitions, ZPE is accounted for in the vibrational partition function. Indeed, the pre-factor v_{des} is given by various partition functions, q, including those in Equations (10-12).

$$q^{gas}_{trans,2D} = A \cdot \frac{2\pi \cdot m \cdot k_B \cdot T}{h^2} \quad \text{(S6)},$$

$$q^{ads/gas}_{vib} = \prod_n \frac{exp\left(-\frac{h \cdot v_n}{2 \cdot k_B \cdot T}\right)}{1 - exp\left(-\frac{h \cdot v_n}{k_B \cdot T}\right)} \quad \text{(S7)},$$

$$q^{gas}_{rot} = \frac{T}{\sigma^{sym} \cdot T_{rot}} \quad \text{(S8)}.$$

The $q^{gas}_{trans,2D}$, q^{gas}_{rot}, and q^{gas}_{vib} refer to the gas phase translational partition function including just 2D degrees of freedom (as the third dimension is the reaction coordinate for desorption), the rotational partition function, and the vibrational partition function, respectively, computed in a large box. The q^{ads}_{vib} is the vibrational partition function of the adsorbed molecule where six degrees of freedom correspond to frustrated rotations and translations, *vide supra*. Finally, for the rotational partition functions, see Eq. (S8), T_{rot} is the rotational temperature of the adsorbed species.

For the reactive and diffusive steps, the corresponding rates, r_j, have been obtained as well by TST,[4] defined as:

$$r_j = v \cdot exp\left(-\frac{\Delta E_{TS}}{k_B \cdot T}\right);\ v = \frac{k_B \cdot T}{h}\frac{q_{vib}^{TS}}{q_{vib}^{IS}} \quad \text{(S9)},$$

where ΔE_{TS} represents the non-ZPE corrected energy barrier, the pre-factor for v can be determined by the partition function, which refers to the vibrational partition functions in the initial state (IS) or the transition state (TS) on the surface; $q_{vib}^{TS/IS}$ denotes the vibrational partition given by:

$$q_{vib}^{TS/IS} = \prod_n \frac{exp\left(-\frac{h \cdot v_n}{2 \cdot k_B \cdot T}\right)}{1 - exp\left(-\frac{h \cdot v_n}{k_B \cdot T}\right)} \quad \text{(S10)}.$$

Section S3: The Span Model

The energy span model has been widely used to assess activity beyond traditional methods, which consider all individual transition states.[5,6] Within the span model, the rate-determining transition state (RDTS) is identified as the transition state with the highest energy, which influences the reaction rate significantly. The rate-determining intermediate (RDI), i.e., the one with the lowest energy, is used to seize the energy span.[7] The span energy barrier, E_b^{span}, captures the energetic requirement of the reaction, defined as:

$$E_b^{span} = E_{highest}^{RDTS} - E_{lowest}^{RDI} \quad \text{(S11).}$$

Further details on the applicability of the span model are found in the literature.[7,8]

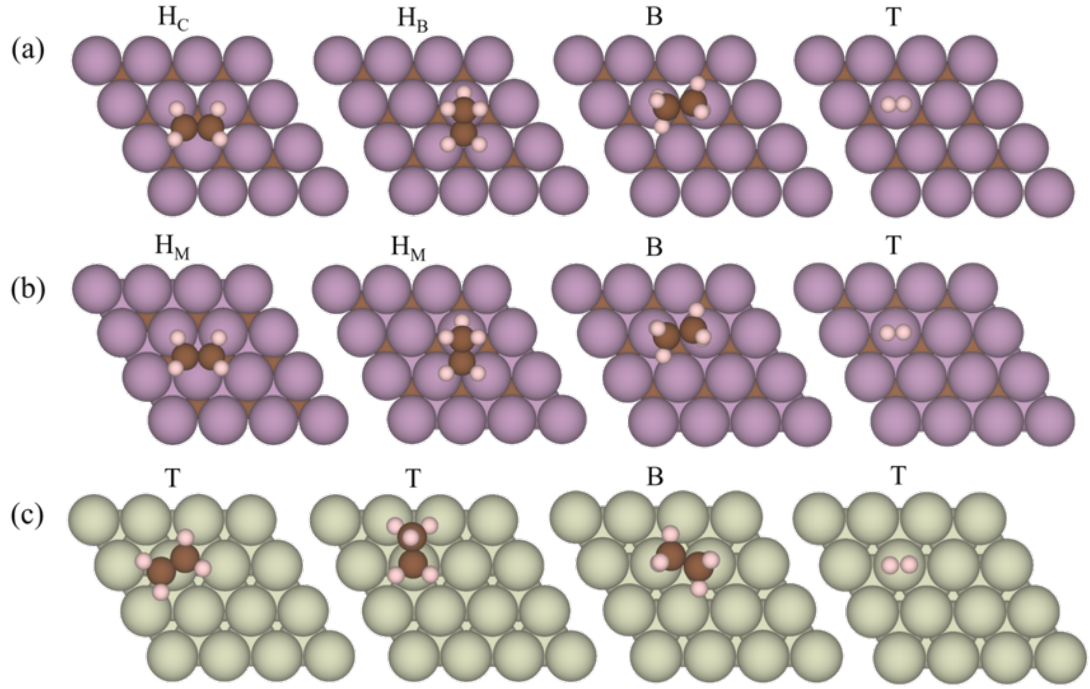

Figure S2. Top views of the lowest-energy high-symmetry surface sites of $C_2H_4^*$, $C_2H_5^*$, $C_2H_6^*$, and H_2^* species on (a) ABA-Mo_2C (0001), (b) ABC-Mo_2C (0001), and (c) Rh (111). Notations above the calculated structures denote the most stable adsorption site, being top (T), bridge (B), or hollow sites with a C underneath (H_C) or a metal (H_M), or being a mixed bridge sites (H_B).

Table S1. Adsorption Gibbs free energies of H_2, C_2H_4, and C_2H_6 on ABA and ABC-Mo_2C (0001) surfaces, and on Rh (111) surface, at the experimental reaction conditions of 1 bar gas partial pressure, *p*, and a temperature, *T*, of 250 °C for Mo_2C MXenes, and 60 °C for Rh. All values are given in eV.

ΔG^{ad} (T, p)	H_2	C_2H_4	C_2H_6
ABA-Mo_2C	−1.64	−1.70	−0.39
ABC-Mo_2C	−1.87	−2.09	−0.40
Rh	−1.15	−1.31	−0.43

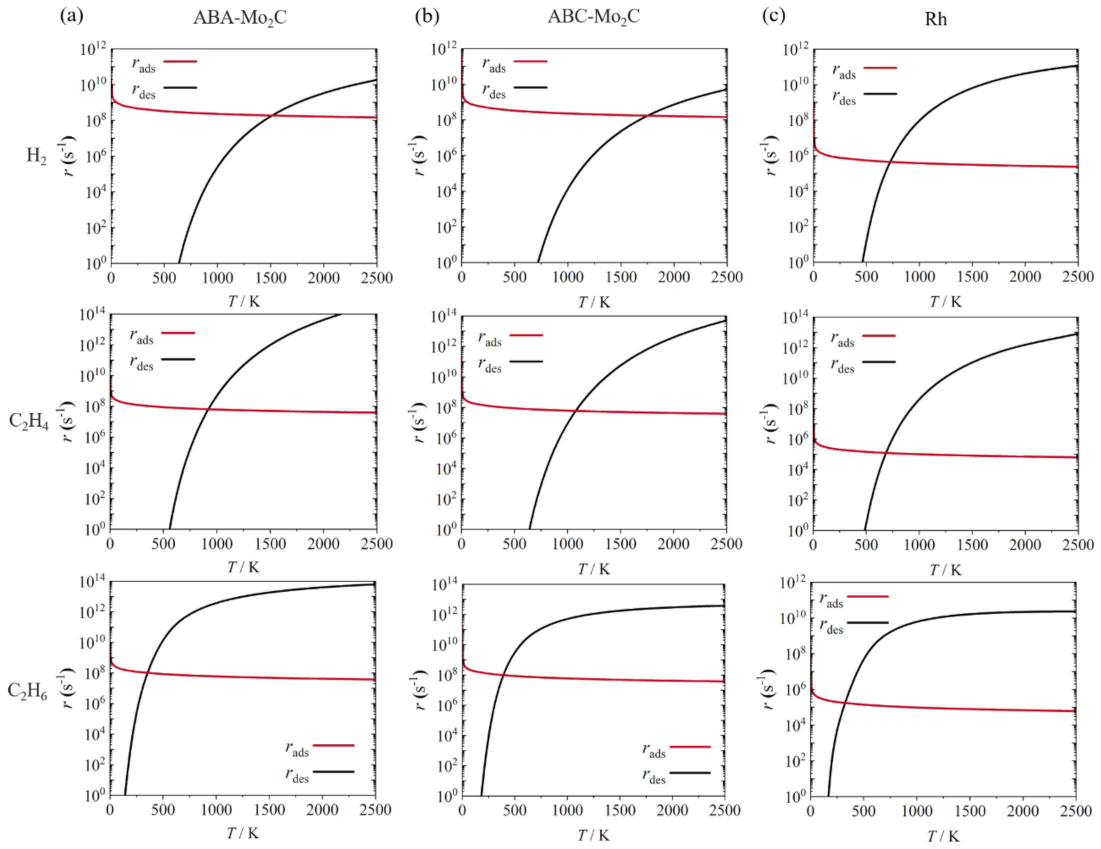

Figure S3. Calculated rates of adsorption, r_{ads}, and desorption, r_{des}, of H_2, C_2H_4, and C_2H_6 on (a) ABA-Mo_2C, (b) ABC-Mo_2C, and (c) Rh (111) models as a function of temperature, *T,* at the total gas pressure of 1 bar.

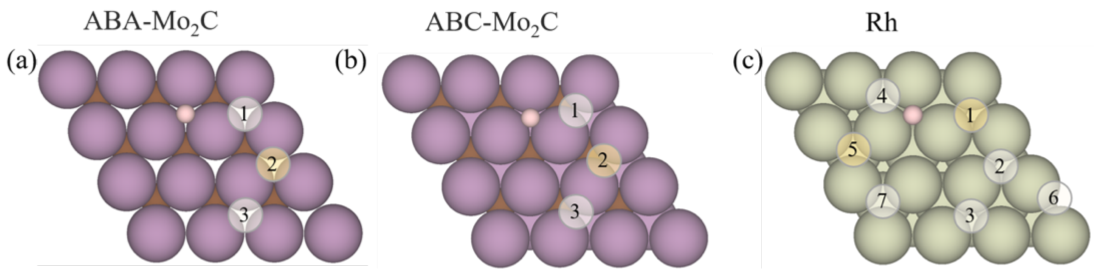

Figure S4. The lowest-energy high-symmetry surface sites of two vicinal H adatoms on (a) ABA-Mo_2C and (b) ABC-Mo_2C (0001) surfaces, and (c) Rh (111) surface. Numbered circles represent the potential high-symmetry positions for the second H adatom, while orange circles represent the final most stable positions.

Table S2. Adsorption energies of two H adatoms on ABA- and ABC-Mo_2C and Rh surfaces on different sites as specified in Figure S4. All values are given in eV, and do not include the ZPE term. The bold font represents the lowest energy case.

	1	2	3	4	5	6	7
ABA-Mo_2C	−1.73	**−1.75**	−1.72	—	—	—	—
ABC-Mo_2C	−1.93	**−1.97**	−1.95	—	—	—	—
Rh	**−1.19**	−1.19	−1.18	−0.95	**−1.16**	−1.17	−1.16

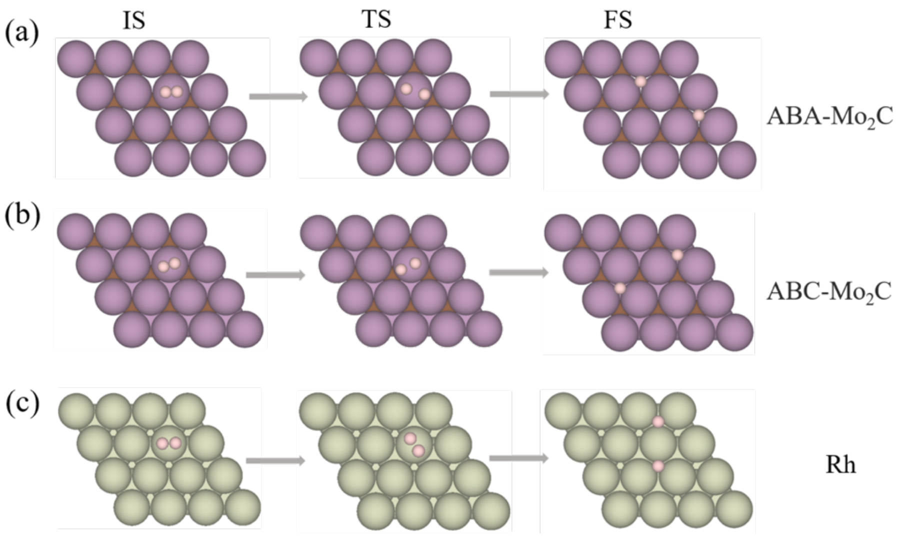

Figure S5. Top view of the H_2 dissociation on (a) ABA-Mo_2C, (b) ABC-Mo_2C, and (c) Rh (111) pristine surfaces, including initial states (ISs), transition states (TSs), and final states (FSs).

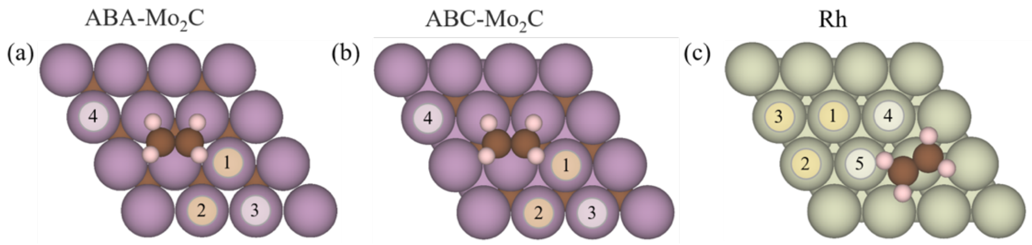

Figure S6. The lowest-energy co-adsorption sites of H_2 nearby of the adsorbed C_2H_4* on (a) ABA-Mo_2C, (b) ABC-Mo_2C, and (c) Rh (111) surfaces. Numbered circles represent potential high-symmetry positions for the co-adsorption of H_2, while orange circles represent the final most stable positions.

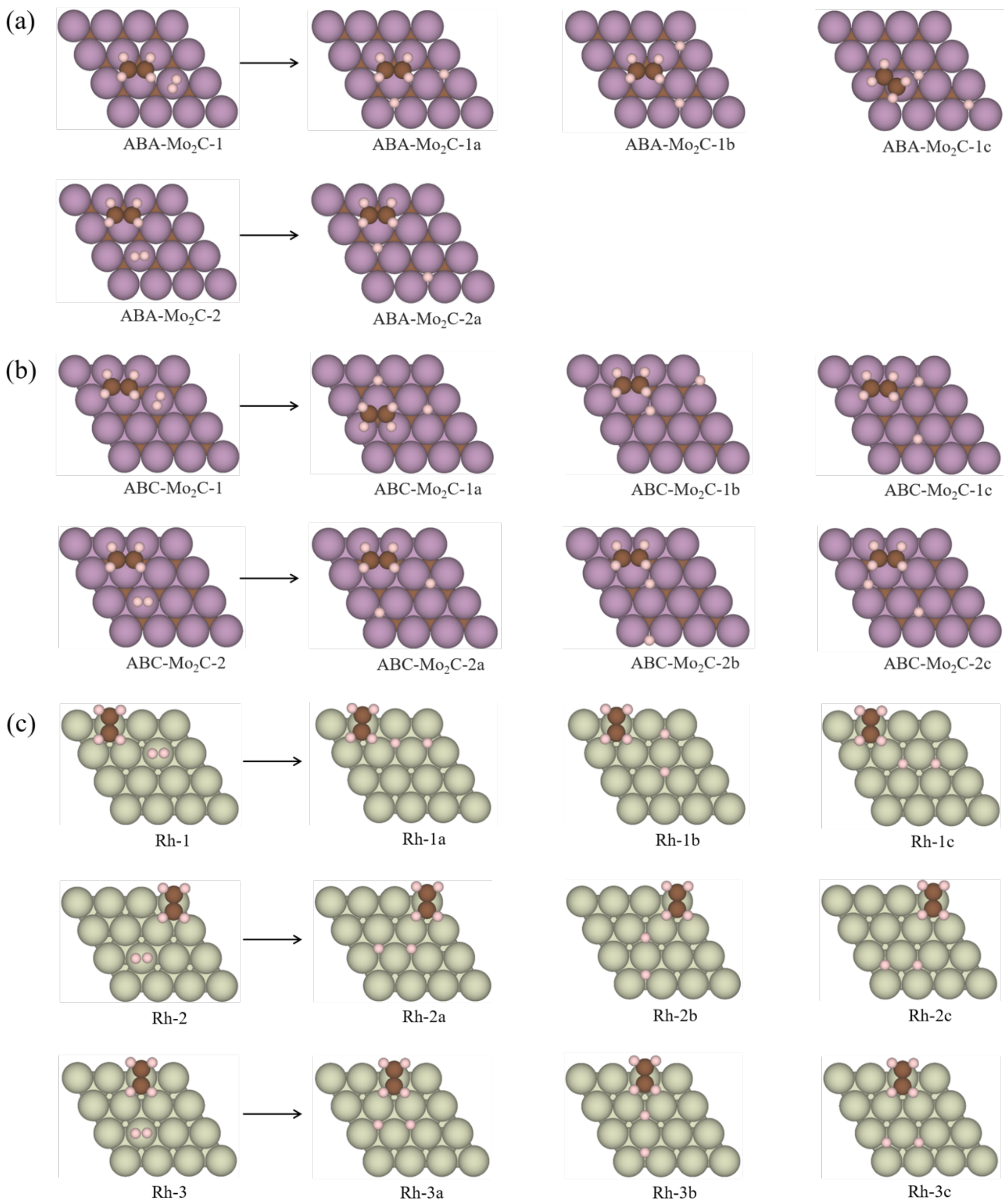

Figure S7. The main high-symmetry surface sites for the H_2 adsorption and two vicinal H* adatoms produced as a result of the H_2 dissociation on (a) ABA-Mo_2C-1 and ABA-Mo_2C-2, (b) ABC-Mo_2C-1 and ABC-Mo_2C-2, and (c) Rh-1, Rh-2, and Rh-3 surfaces in the presence of C_2H_4. For details on the notations used, see Figure S6.

Table S3. H_2 adsorption energies on ABA-Mo_2C, ABC-Mo_2C, and Rh surface models with different relative sites with respect to pre-adsorbed C_2H_4 as specified in Figure S6. All values are given in eV. The bold font represents the ones selected to study H_2 dissociation forward.

	1	2	3	4	5
Mo_2C_{ABA}	**−0.42**	**−0.55**	−0.51	−0.55	—
Mo_2C_{ABC}	**−0.67**	**−0.77**	−0.72	−0.76	—
Rh	**−0.52**	**−0.53**	**−0.53**	—	—

Table S4. Total adsorption energies of two H adatoms on ABA-Mo_2C (0001) surface on different sites with respect to pre-adsorbed C_2H_4 as specified in Figure S7a. All values are given in eV. The bold font represents the one selected on the study.

	a	b	c
ABA-Mo_2C-1	**−1.67**	**−1.66**	−1.54
ABA-Mo_2C-2	**−1.64**	—	—

Table S5. Total adsorption energies of two H adatoms on ABC-Mo_2C (0001) surface on different sites with respect to pre-adsorbed C_2H_4 as specified in Figure S7b. All values are given in eV. The bold font represents the one selected on the study.

	a	b	c
ABC-Mo_2C-1	−1.84	−1.61	**−1.77**
ABC-Mo_2C-2	**−1.91**	−1.59	−1.61

Table S6. Total adsorption energies of two H adatoms on Rh (111) surface on different sites with respect to pre-adsorbed C_2H_4 as specified in Figure S7c. All values are given in eV. The bold font represents the one selected on the study.

	a	b	c
Rh-1	**−1.11**	**−1.11**	−1.09
Rh-2	**−1.13**	**−1.11**	−1.08
Rh-3	**−1.13**	−1.07	−1.08

Table S7. The energy barriers, E_b, for H_2 dissociation on ABA- and ABC-Mo_2C (0001) surface models, and on Rh (111) surface, according to the ISs and FSs depicted in Figures S7 and S8. All values are given in eV. The bold font represents the H_2 dissociating path with the lowest E_b.

	a	b	c
ABA-Mo_2C-1	0.20	**0.19**	—
ABA-Mo_2C-2	—	0.25	—
ABC-Mo_2C-1	—	—	**0.09**
ABC-Mo_2C-2	0.10	—	—
Rh-1	0.01	0.01	—
Rh-2	0.01	**0.01**	—
Rh-3	0.01	—	—

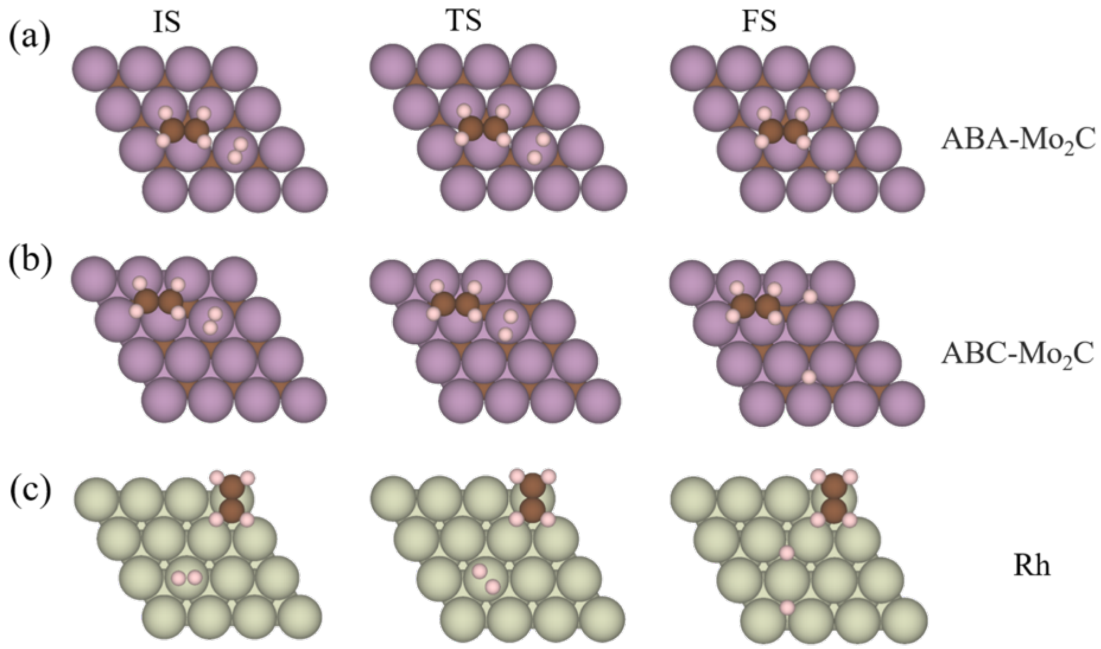

Figure S8. Top views of the different stages of H_2 dissociation on (a) ABA-Mo_2C, (b) ABC-Mo_2C, and (c) Rh (111) surfaces in the presence of C_2H_4*, including initial states (ISs), transition states (TSs), and final states (FSs) with the lowest energy barriers (E_b, see Table S7).

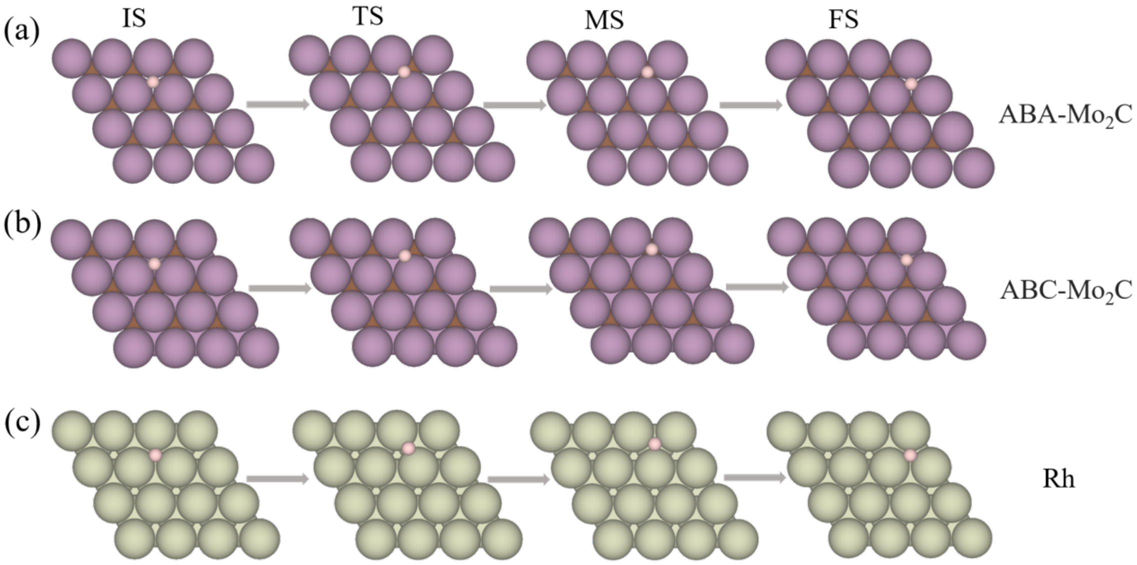

Figure S9. Top views of the different stages of H* diffusion on (a) ABA-Mo_2C, (b) ABC-Mo_2C, and (c) Rh (111) pristine surfaces, including the initial states (ISs), transition states (TSs), middle states (MSs), and final states (FSs).

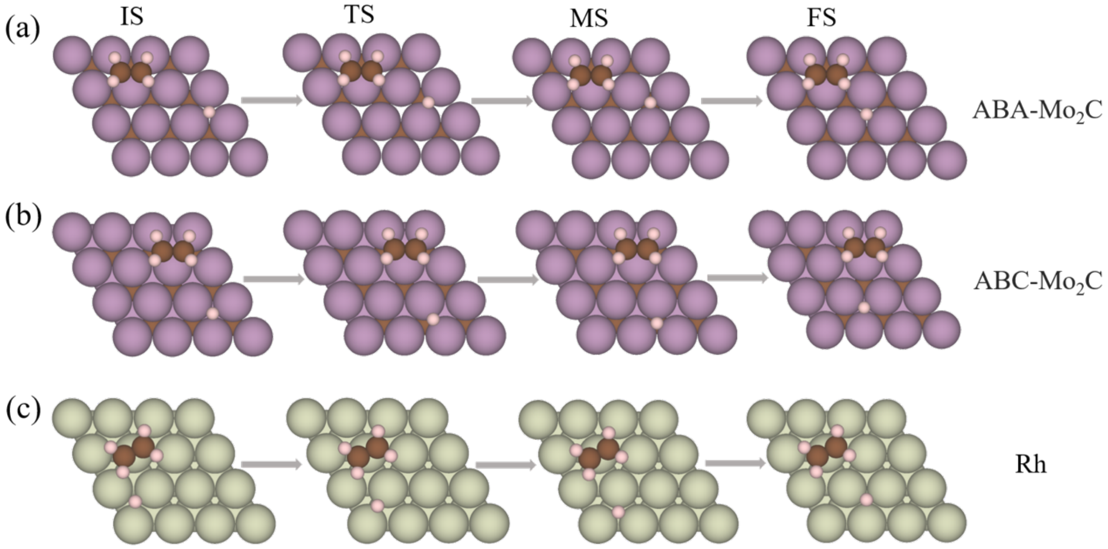

Figure S10. Top views of the different stages of H* diffusion on (a) ABA-Mo_2C, (b) ABC-Mo_2C, and (c) Rh (111) surfaces in the presence of C_2H_4*, including initial states (ISs), transition states (TSs), middle states (MSs), and final states (FSs).

Table S8. Energy barriers, E_b, for H diffusion and H_2 dissociation on ABA-Mo_2C, ABC-Mo_2C, and Rh surfaces, when pristine or in the presence C_2H_4, plus the H_2 adsorption energies. All values are given in eV.

	Pristine			With C_2H_4		
	$E_b^{H\ diff}$	$E_b^{H_2\ diss}$	$E_{ads}^{H_2}$	$E_b^{H\ diff}$	$E_b^{H_2\ diss}$	$E_{ads}^{H_2}$
ABA-Mo_2C	0.35	0.28	−0.53	0.37	0.19	−0.55
ABC-Mo_2C	0.27	0.11	−0.72	0.28	0.09	−0.77
Rh	0.16	0.06	−0.61	0.10	0.01	−0.53

Table S9. Reaction energies, ΔE, and energy barriers, E_b, of the first and second hydrogenation reaction steps on C_2H_4, as well as the full ΔE and the energy barrier according to the span model, E_b^{span}, on the ABA- and ABC-Mo_2C surface models depicted in Figure 3 of the main text, and on Rh (111). All values are given in eV.

	$\Delta E_H^{1^{st}}$	$E_b^{H-1^{st}}$	$\Delta E_H^{2^{nd}}$	$E_b^{H-2^{nd}}$	ΔE_{full}	E_b^{span}
ABA-Mo_2C-1b@1	0.28	0.73	0.92	2.03	1.20	2.30
ABA-Mo_2C-1b@2	0.33	0.79	0.87	1.90	1.20	2.22
**ABA-Mo_2C-1b@3**	**0.29**	**0.74**	**0.91**	**1.77**	**1.20**	**2.06**
ABA-Mo_2C-1b@4	0.34	0.84	0.86	2.07	1.20	2.40
ABC-Mo_2C-1c@1	0.38	0.64	1.32	1.98	1.70	2.36
**ABC-Mo_2C-1c@2**	**0.57**	**0.81**	**1.13**	**1.83**	**1.70**	**2.40**
ABC-Mo_2C-1c@3	0.50	0.78	1.31	2.11	1.70	2.51
**Rh_2b**	**0.39**	**0.91**	**-0.11**	**0.55**	**0.27**	**0.94**

Table S10. Reaction energies, Δ*E*, and energy barriers, E_b, of the first and second hydrogenation reaction steps on C_2H_4, as well as the full ΔE^{span} and the energy barrier according to the span model, E_b^{span}, on the ABA- and ABC-Mo_2C surface models, and on Rh (111) surface, either on their pristine models, or having ¾ ML of H or C_2H_4 depicted in Figures S11-S13.

		$\Delta E_H^{1^{st}}$	$E_b^{H-1^{st}}$	$\Delta E_H^{2^{nd}}$	$E_b^{H-2^{nd}}$	ΔE^{span}	E_b^{span}
Clean	ABA-Mo_2C-1b@3	0.29	0.74	0.91	1.77	1.20	2.06
	ABC-Mo_2C-1c@2	0.57	0.81	1.13	1.83	1.70	2.40
	Rh-2b	0.39	0.91	-0.11	0.55	0.27	0.94
¾ ML H	ABA-Mo_2C-1b@3	0.44	0.78	0.32	1.13	0.76	1.57
	ABC-Mo_2C-1c@2	0.48	0.83	0.62	1.57	1.09	2.05
	Rh-2b	0.33	0.82	-0.13	0.48	0.20	0.80
¾ ML C_2H_4	ABA-Mo_2C-1b@3	0.21	0.67	0.65	1.95	0.85	2.16
	ABC-Mo_2C-1c@2	0.52	0.84	0.78	1.35	1.30	1.87
	Rh-2b	0.32	0.75	-0.04	0.59	0.28	0.91

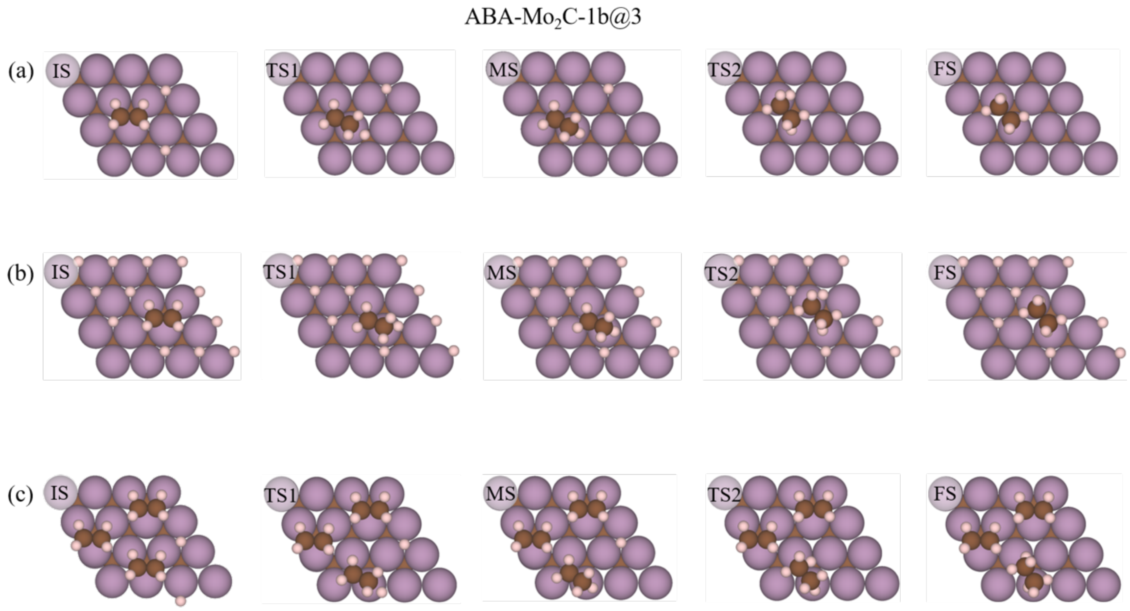

Figure S11. Top views of the IS, MS, FS of C_2H_4 hydrogenation on ABA-Mo_2C (0001) surface, including first (TS1) and second (TS2) hydrogenation transition states on (a) pristine surface, (b) ¾ ML H*, and (c) ¾ ML C_2H_4* models.

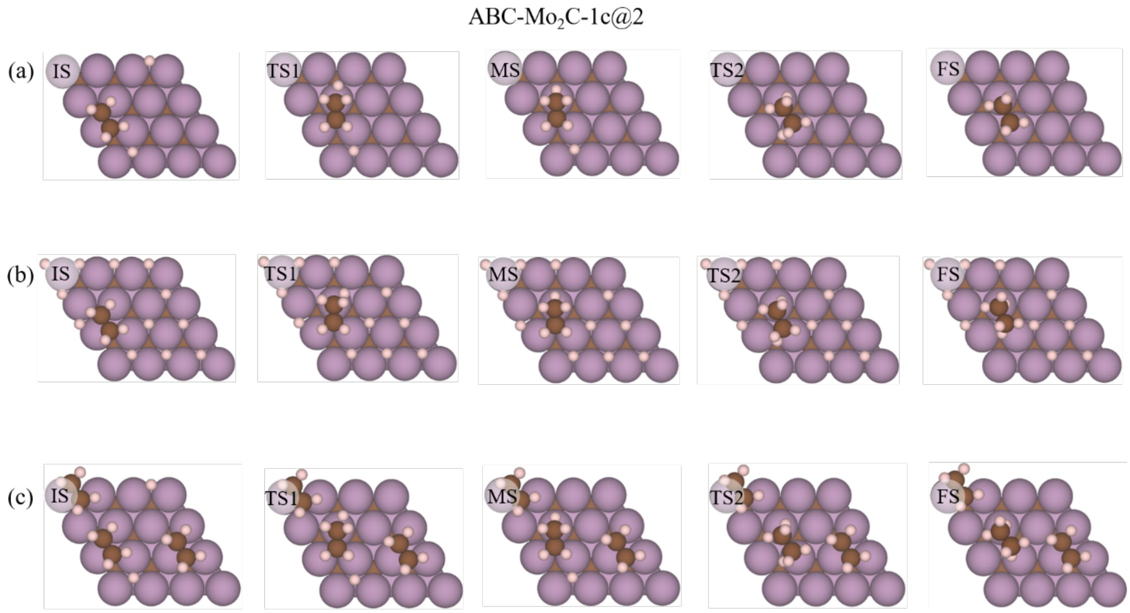

Figure S12. Top views of the IS, MS, FS of C_2H_4 hydrogenation on ABC-Mo_2C (0001) surface, including first (TS1) and second (TS2) hydrogenation transition states on (a) pristine surface, (b) ¾ ML H*, and (c) ¾ ML C_2H_4* models.

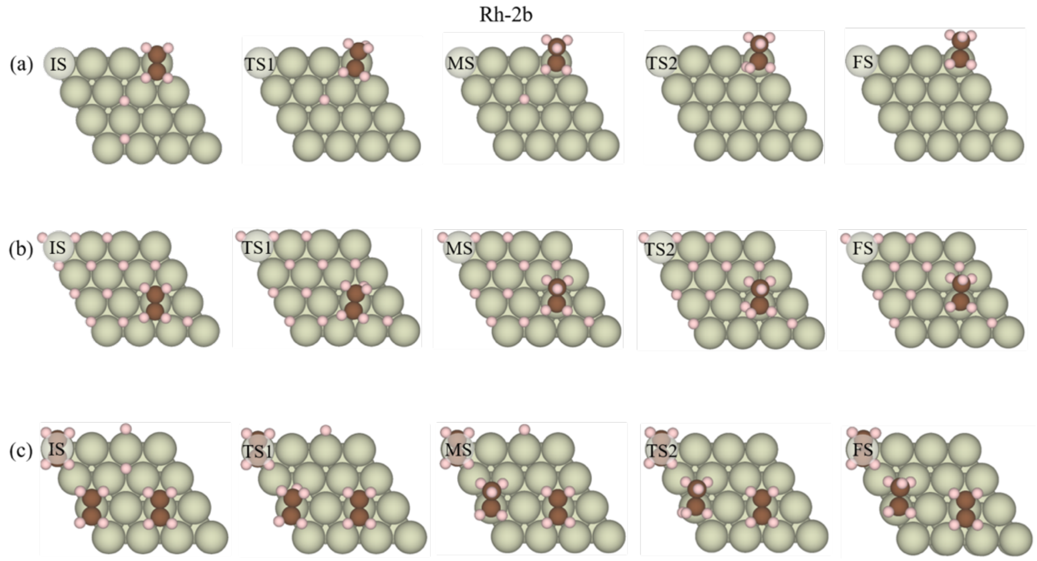

Figure S13. Top views of the IS, MS, FS of C_2H_4 hydrogenation on Rh (111) surface, including first (TS1) and second (TS2) hydrogenation transition states on (a) pristine surface, (b) ¾ ML H*, and (c) ¾ ML C_2H_4* models.

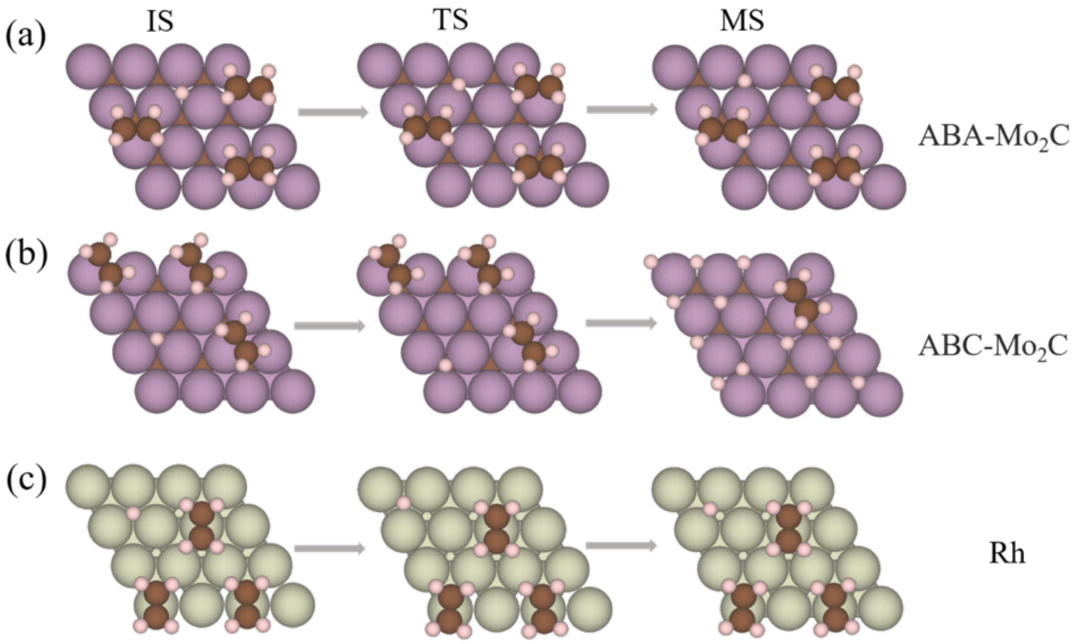

Figure S14. Top views of the IS, TS, and MS of H diffusion on (a) ABA-Mo_2C, (b) ABC-Mo_2C, and (c) Rh (111) surface models with ¾ ML of C_2H_4*. Notice that because of symmetry, the final diffusion from MS to FS is the same as from IS to MS.

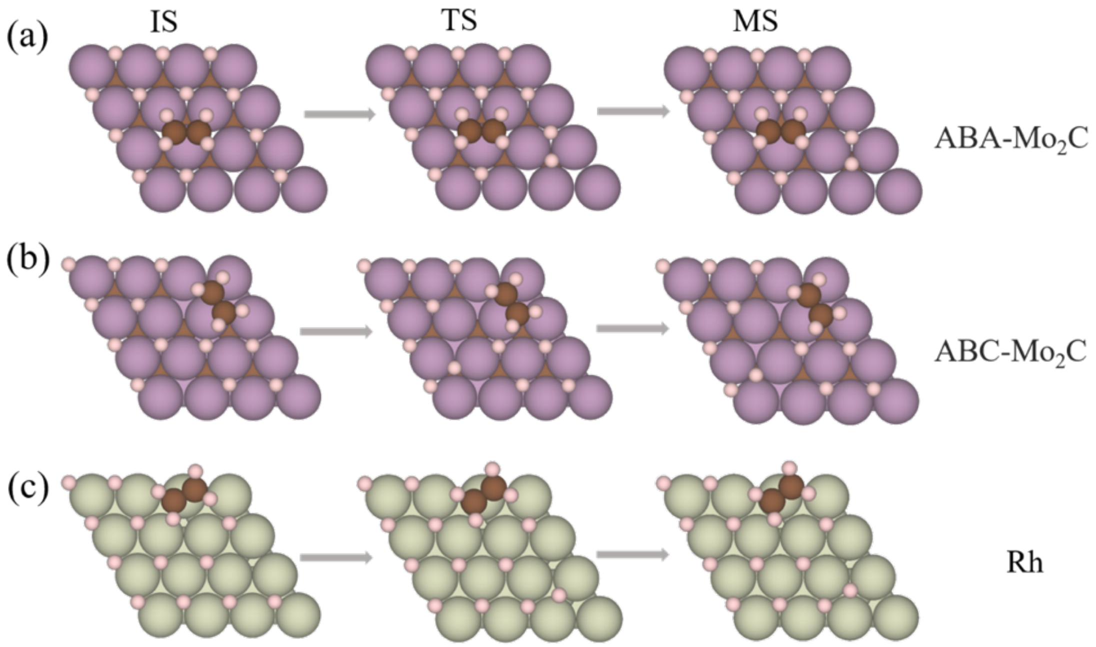

Figure S15. Top views of the IS, TS, and MS of H diffusion on (a) ABA-Mo_2C, (b) ABC-Mo_2C, and (c) Rh (111) surface models with ¾ ML of H*. Notice that because of symmetry, the final diffusion from MS to FS is the same as from IS to MS.

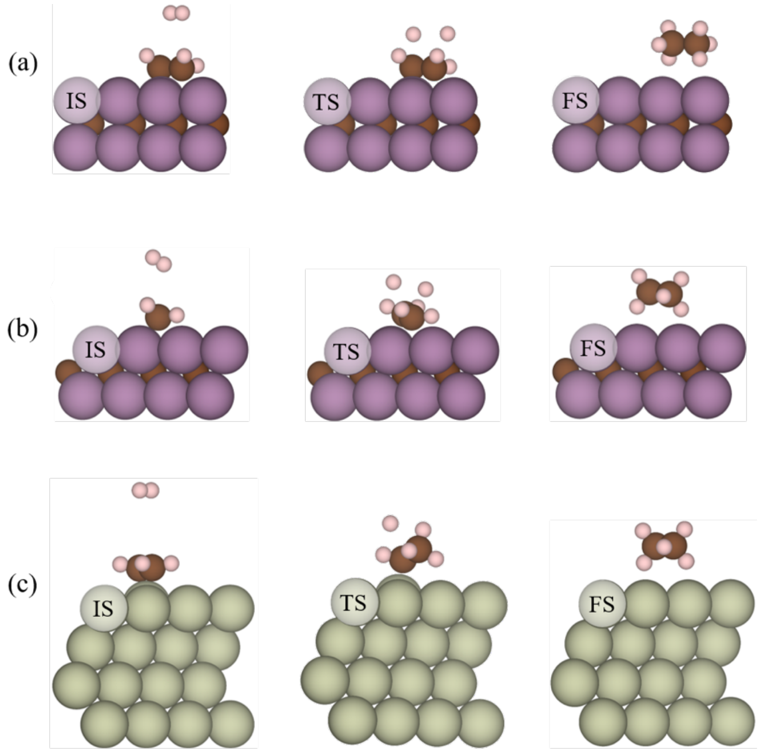

Figure S16. Side views of the IS, TS, and FS of Eley-Rideal mechanism on (a) ABA-Mo_2C, (b) ABC-Mo_2C, and (c) Rh (111) pristine surface models.

Table S11. Energy barriers, E_b, for H diffusion on ABA-Mo_2C, ABC-Mo_2C, and Rh surfaces, either on their pristine models, or having ¾ ML of H or C_2H_4 depicted in Figure 4. All values are given in eV.

	Clean	¾ ML H	¾ ML C_2H_4
ABA-Mo_2C	0.37	0.42	0.36
ABC-Mo_2C	0.28	0.42	0.27
Rh	0.10	0.16	0.11

Table S12. Conversion of propene, $X_{C_3H_6}$, and NMR signal enhancement, SE, for the CH_3-groups of propane obtained in the hydrogenation of propene with p-H_2 over Mo_2CT_{x-500} and Rh/TiO_2 catalysts at different temperatures (see the experimental section for details).

Catalyst	*T*, °C	Flow rate, mLs min^{-1}	X_{C3H6}, %	*SE*
Mo_2CT_{x-500}	165	26	100	–
		155	86	–
		240	75	–
	205	26	100	–
		156	82	–
		240	73	1.1
	235	26	100	–
		156	79	1.9
		240	62	2
	275	26	100	–
		156	60	–
		240	49	4
	325	26	97	–
		156	42	1.3
		240	32	5
	375	26	74	–
		240	9	10
Rh/TiO_2	43	26	43	–
		156	11	161
		240	5	337
	53	26	52	–
		156	12	169
		240	7	347
	60	26	61	–
		156	20	154
		240	11	331
	70	26	72	–
		156	23	154
		240	14	352
	85	26	94	–
		156	32	143
		240	22	316
	100	26	100	–
		156	38	145
		240	28	318
	125	26	100	–

		156	47	131
		240	36	297
	150	26	100	–
		156	53	127
		240	40	298

References

(1) Gilson, M. K.; Irikura, K. K. Symmetry Numbers for Rigid, Flexible, and Fluxional Molecules: Theory and Applications. *J. Phys. Chem. B* **2010**, *114*, 16304–16317.

(2) "Collision Theory." Chemistry LibreTexts, Libretexts, 22 May 2017.

(3) Kunkel, C.; Viñes, F.; Illas, F. Transition Metal Carbides as Novel Materials for CO_2 Capture, Storage, and Activation. *Energy Environ. Sci.* **2016**, *9*, 141–144.

(4) Truhlar, D. G.; Garrett, B. C.; Klippenstein, S. J. Current Status of Transition-State Theory. *J. Phys. Chem.* **1996**, *100*, 12771–12800.

(5) Guiducci, A. E.; Boyd, C. L.; Clot, E.; Mountford, P., Reactions of Cyclopentadienyl-Amidinate Titanium Imido Compounds with CO_2: Cycloaddition-Extrusion *vs.* Cycloaddition-Insertion. *Dalton Trans.* **2009**, *30*, 5960–5979.

(6) Tukov, A. A.; Normand, A. T.; Nechaev, M. S., N-Heterocyclic Carbenes Bearing Two, One and No Nitrogen Atoms at the Ylidenecarbon: Insight from Theoretical Calculations. *Dalton Trans.* **2009**, *30*, 7015–7028.

(7) Kozuch, S.; Shaik, S., How to Conceptualize Catalytic Cycles? The Energetic Span Model. *Acc. Chem. Res.* **2011,** *44*, 101–110.

(8) Kozuch, S., A Refinement of Everyday Thinking: the Energetic Span Model for Kinetic Assessment of Catalytic Cycles. *Comput. Mol. Sci.* **2012,** *2*, 795–815.
